# Total synthesis of panicein A_2_

**DOI:** 10.3762/bjoc.11.215

**Published:** 2015-10-26

**Authors:** Lili Yeung, Lisa I Pilkington, Melissa M Cadelis, Brent R Copp, David Barker

**Affiliations:** 1 School of Chemical Sciences, University of Auckland, 23 Symonds Street, Auckland, New Zealand

**Keywords:** modified Claisen rearrangement, sesquiterpene, chromenol, total synthesis

## Abstract

The first total synthesis of the unusual aromatic sesquiterpene panicein A_2_ is reported and the structure of the natural product has been confirmed. When tested by the NCI against a range of human cancer cell lines, it was found that panicein A_2_ exhibits very little antiproliferative activity at 10 μM – an observation that is at odds with the earlier report that stated panicein A_2_ exhibits in vitro cytotoxicity against a number of tumour cell lines.

## Introduction

**Figure 1:**
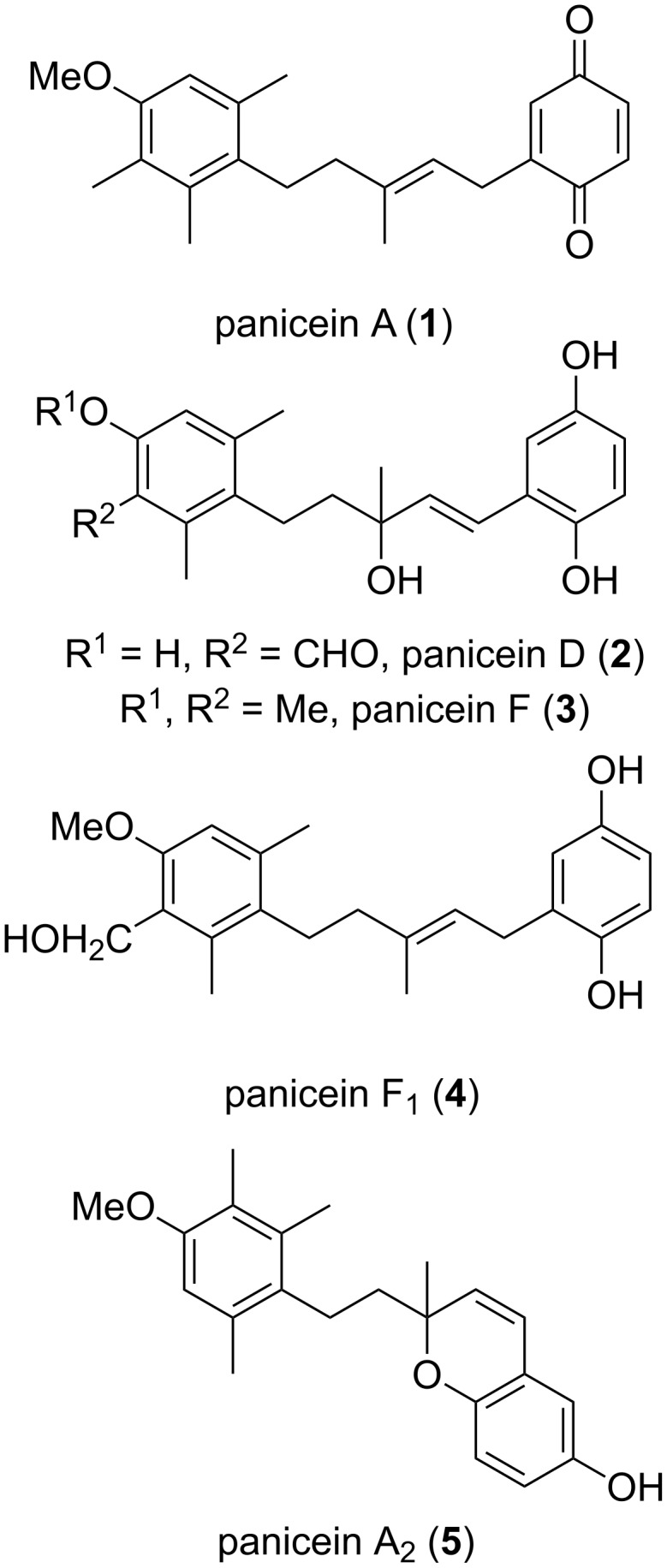
Members of the panicein family of aromatic sesquiterpenoids.

The panicein family is an unusual family of natural products, which generally consist of an aromatic sesquiterpene group linked to a quinone (as seen in panicein A (**1**), Figure 1), hydroquinone moiety (as seen in paniceins D (**2**), F (**3**) and F_1_ (**4**)) or chromenol as seen in panicein A_2_ (**5**).

**Figure 2:**
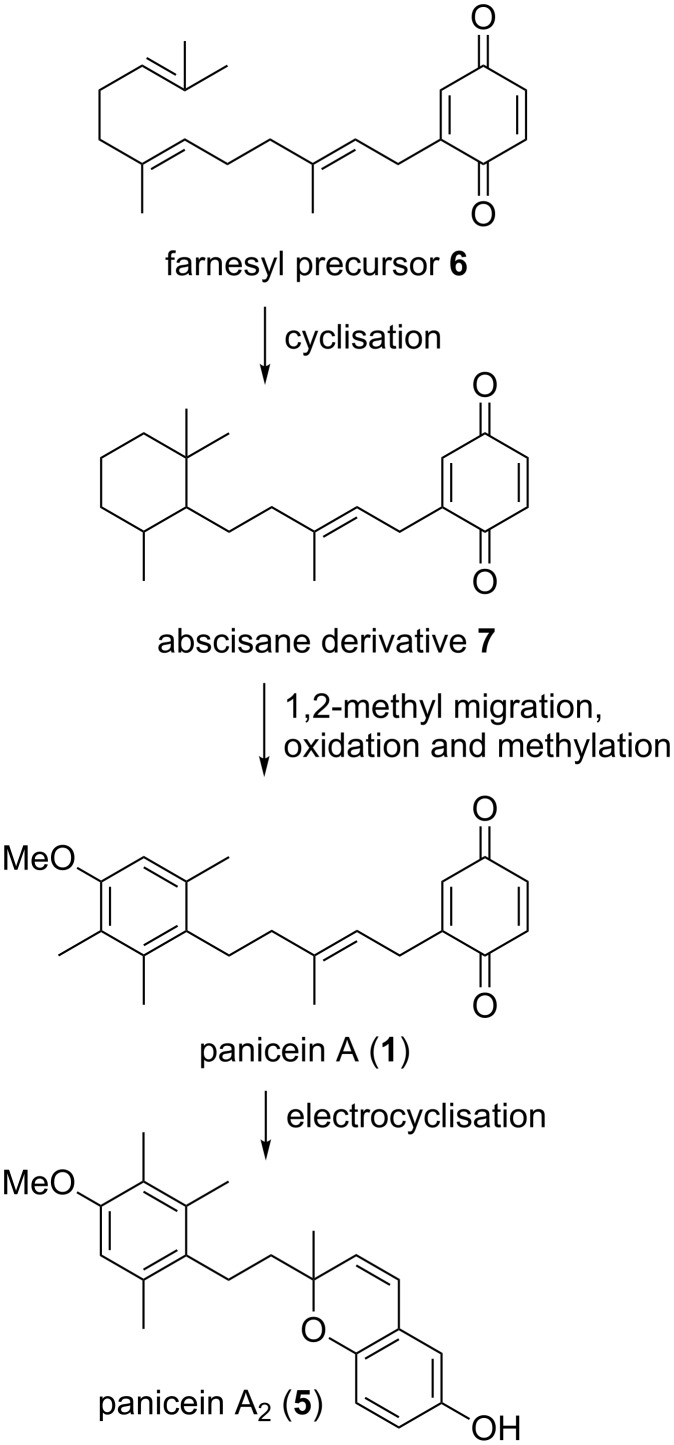
Proposed biogenesis of panicein A_2_ (**5**).

The first members of the panicein family were isolated by Cimino et al. in 1973 from the marine sponge *Halichondria panacea* [1]; members of this family have since been isolated from *Reniera fulva* and *R. mucosa* [2–3]. It has been postulated that the biosynthesis of paniceins centres around the cyclisation of a farnesyl precursor **6** [4] to an abscisane derivative **7** followed by a 1,2-methyl migration and subsequent oxidation to give panicein A (**1**) [1,3]. Subsequent electrocyclisation [5–6] would afford panicein A_2_ (**5**) (Figure 2). This hypothesis has been supported by the isolation of species believed to be intermediates along this proposed reaction pathway [3].

Panicein A_2_ (**5**), an example of a cyclised variant of the panicein structure, is a racemic compound that was first isolated in 1994 from *Reniera mucosa* alongside its non-cyclised isomer **1**, and ten other members of the panicein family [3]. Panicein A_2_ (**5**) and D (**2**) were reported to exhibit in vitro cytotoxicity against four cancer cell lines (P388 mice lymphoma, A549 human lung carcinoma, HT29 human colon carcinoma and MEL28 human melanoma) with an ED_50_ of 5 μg/mL [3]. Panicein F_1_ (**4**) was active against P388, A549 and MEL20 cell lines (ED_50_ = 2.5 μg/mL). The diversity of antiproliferative activities shown by these compounds, and particularly panicein A_2_ (**5**), presents them to be attractive synthetic targets.

**Figure 3:**
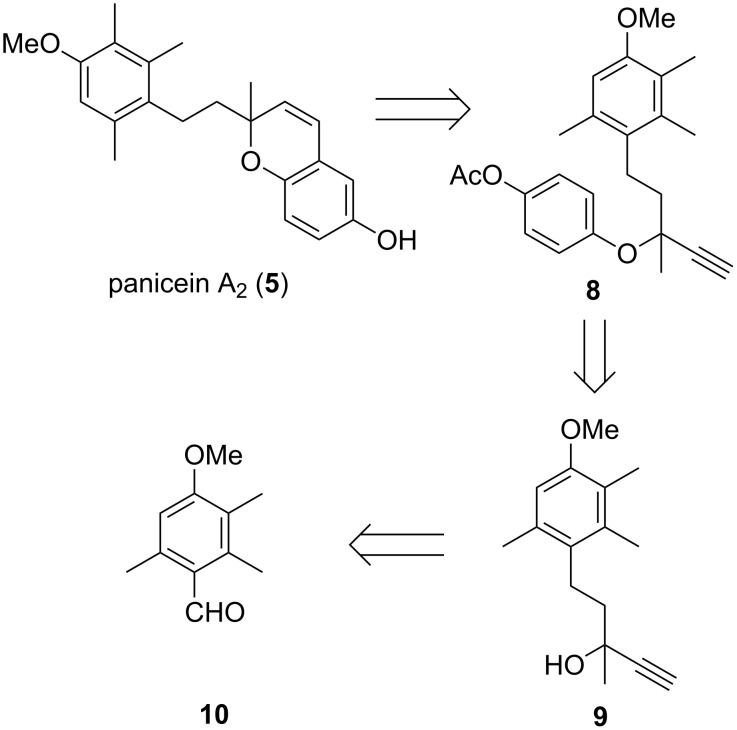
Retrosynthetic analysis of panicein A_2_ (**5**).

We proposed that panicein A_2_ (**5**) could be synthesised from the cyclisation and subsequent deprotection of propargyl ether **8**. Ether **8** could be formed through the addition of the appropriate phenol to acetylenic alcohol **9**, which itself can be derived from aldehyde **10** (Figure 3).

## Results and Discussion

### Synthesis of panicein A_2_

**Scheme 1:**
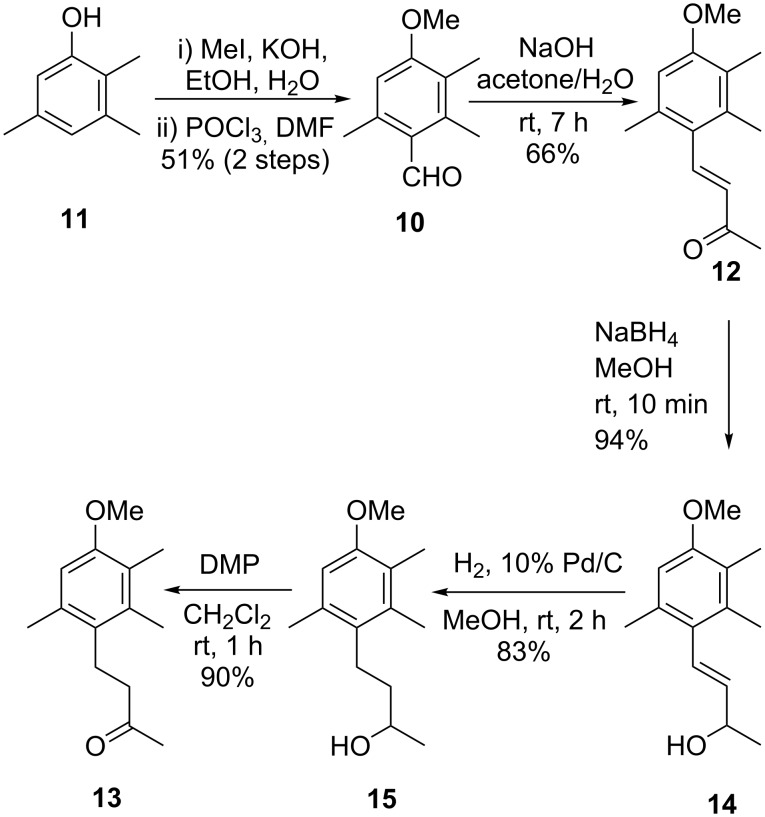
Synthesis of ketone **13**.

Firstly, aldehyde **10** was required; it was prepared through methylation followed by Vilsmeier–Haack reaction of 2,3,5-trimethylphenol (**11**), giving **10** in 51% yield over two steps (Scheme 1) [7]. Aldehyde **10** then underwent an aldol reaction according to the procedures of Samokhvalov et al. to provide ketone **12** in 66% yield [8]. The selective reduction of the olefin in α,β-unsaturated ketone **12** was then attempted using the Raney nickel catalyst under hydrogen as previously reported [8]; unfortunately no product **13** was formed. Following this, a range of conditions were employed to reduce the double bond in the presence of the α,β-unsaturated ketone, including Pd/C and H_2_, NaBH_4_ and Pd/C in the presence of acetic acid [9], and NaBH_4_ with CoCl_2_ [10]; all of these reductive conditions gave complex, inseparable mixtures of overreduction products of the ketone functionality. We therefore decided to selectively reduce the ketone to an alcohol – this would allow for the uncomplicated hydrogenolysis of the olefin. Following this, subsequent oxidation of the alcohol would give the desired ketone **13**. The reduction of ketone **12** to alcohol **14** with NaBH_4_ was complete after a reaction time of 10 minutes, giving **14** in an excellent 94% yield. Hydrogenolysis of the alkene in **14** using Pd/C in MeOH proceeded in 2 hours, providing alcohol **15** which was then oxidised to the desired ketone **13** with Dess–Martin periodinane in 75% yield over two steps. Although this approach was ultimately two steps longer than initially planned, ketone **13** was provided in three short steps from **12**, all of which were high yielding, even when conducted on a multigram scale.

**Scheme 2:**
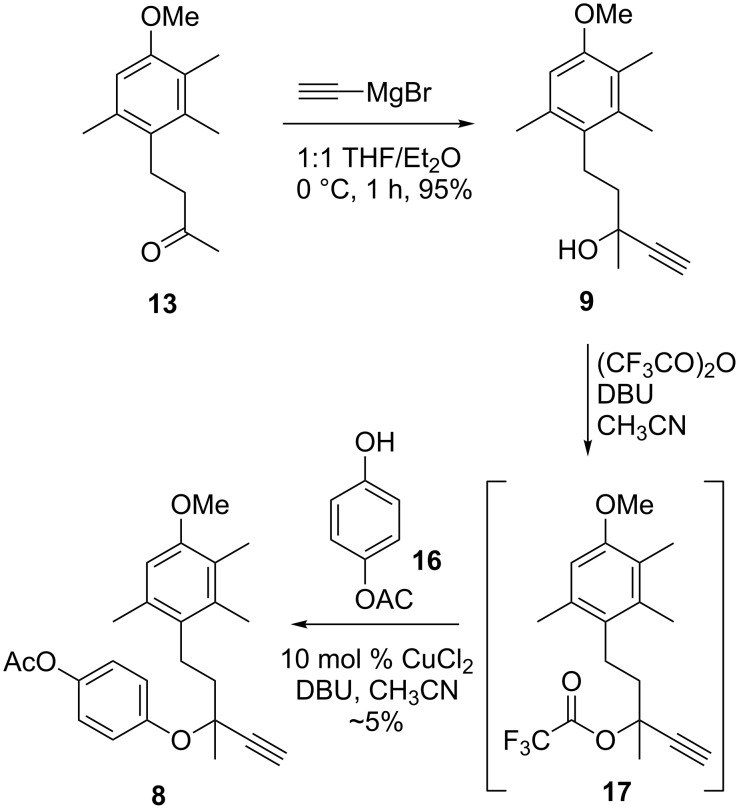
Synthesis of propargyl ether **8** through formation of trifluoroacetate intermediate **17**.

The addition of ethynylmagnesium bromide to ketone **13** was initially attempted using THF as the solvent, only providing alcohol **9** in 47% yield (Scheme 2). Altering the solvent system to a 1:1 mixture of THF and diethyl ether, which also improved the solubility of the Grignard reagent, increased the yield of **9** to 95%. With alcohol **9** in hand, the next step was the key coupling of alcohol **9** and phenol **16** [11] to form the required propargyl ether **8**. Godfrey et al. demonstrated an efficient, one-step method to synthesise propargyl ethers bearing electron withdrawing groups under mild conditions, achieved through the in situ formation of a trifluoroacetate intermediate and subsequent addition of a phenol [12], a method that has since been utilised in a number of syntheses [13–17]. The conversion of alcohol **9** to trifluoroacetate **17** by the addition of trifluoroacetic anhydride and DBU was monitored by TLC and observed to be complete after 1 hour. The phenoxide of phenol **16**, prepared by the deprotonation of **16** by DBU, was then added to intermediate **17**. Unfortunately, the desired product **8** was only obtained in trace amounts.

**Scheme 3:**
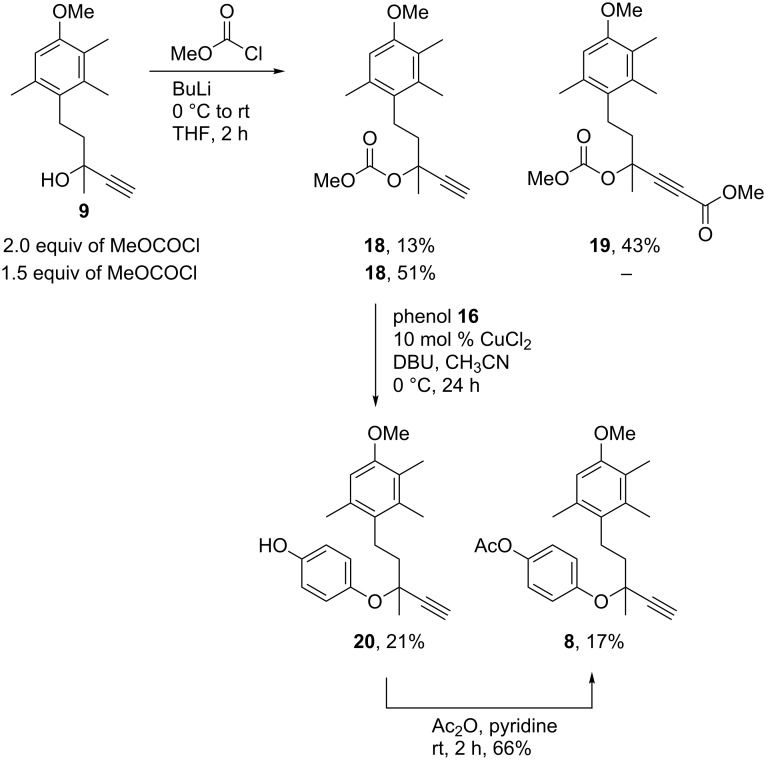
Synthesis of propargyl ether **8** through carbonate **18**.

Carbonate intermediates have been shown to be an effective alternative to trifluoroacetate intermediates [18], and have the added advantage to being stable to an aqueous work-up and thus can be isolated. With that knowledge, it was decided to synthesise carbonate **18** through the addition of methyl chloroformate to alcohol **9** following treatment of **9** with *n*-BuLi. Unfortunately, when the reaction was first attempted using 2 equivalents of methyl chloroformate, only 13% of the desired carbonate was formed, with the major product (43%) being the double-addition species **19** (Scheme 3). Lowering the amount of methyl chloroformate to 1.5 equivalents provided the desired product **18** in 51% yield. Phenol **16** was successfully added to carbonate **18** using the conditions previously attempted, giving a mixture of propargyl ether **8** and its deacetylation product **20**.

**Scheme 4:**
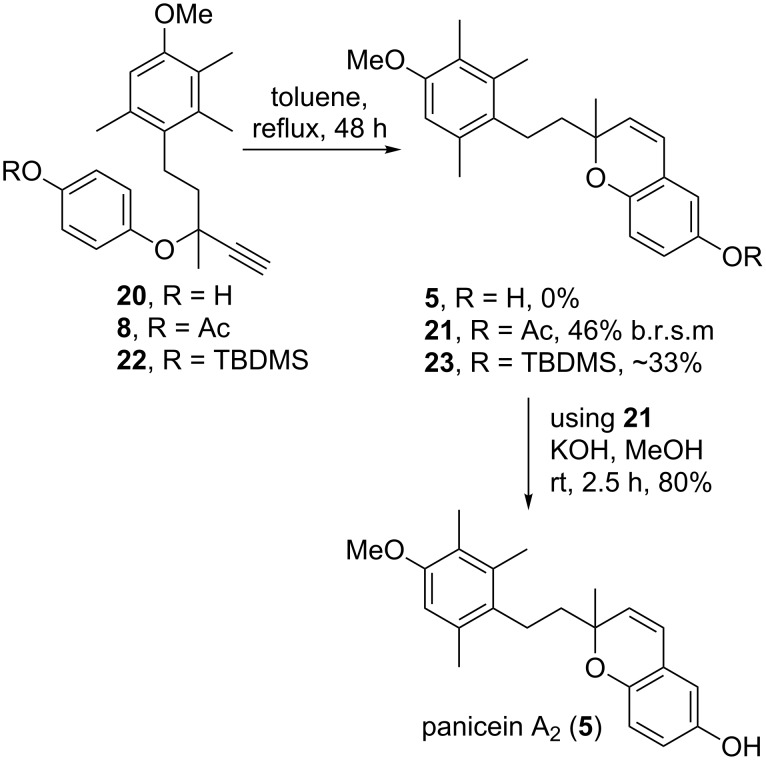
Synthesis of panicein A_2_ (**5**).

The direct conversion of deacetylated product **20** to panicein A_2_ (**5**) through a modified Claisen rearrangement [19–20] was then attempted. Unfortunately, after heating **20** in toluene for 48 hours, no desired product **5** was obtained, with a complex mixture of compounds produced (Scheme 4). The reaction was then attempted with the acetyl-protected propargyl ether **8**. Gratifyingly, cyclised product **21** was obtained in a 46% yield, based on returned starting material. To investigate the effect of an alternative protecting group on the cyclisation reaction, TBDMS ether **22** was synthesised from alcohol **20**. Cyclisation of **22** did proceed to give the desired product **23**, however in a yield of about 33%, further complicated by being inseparable from the starting material **22** (Scheme 4). Due to the poor yields of this reaction, we decided to proceed with the acetate-protected species, and thus alcohol **20** was converted to acetate **8** in 66% yield with acetic anhydride in pyridine (Scheme 3). Finally, acetate **21** was deprotected to give the natural product panicein A_2_ (**5**) in 80% yield. The NMR data for synthetic panicein A_2_ (**5**) matched literature values (see Supporting Information File 1 for data comparison tables).

### Biological testing

When isolated, natural panicein A_2_ (**5**) exhibited activity against four cancer cell lines with ED_50_ = 5 μg/mL ≈ 15 μM. To further expand upon these promising results, synthetic panicein A_2_ (**5**) was tested by the NCI against 57 human cancer cell lines, through their developmental therapeutics program. Interestingly, at the tested concentration of 10 μM, panicein A_2_ (**5**) showed very little activity against most cell lines. The best performance of panicein A_2_ (**5**) was against T-47D, a breast cancer cell line, in which it showed a 43% reduction of growth when compared to a control. Two of the cell lines tested by the NCI were the same as those tested against in the original isolation study (A549 and HT29). Our results show that panicein A_2_ (**5**) only reduces growth of A549 human lung carcinoma by 14% and had no effect on the HT29 human colon cancer cell line compared to control. These results indicated panicein A_2_ (**5**) to have poor antiproliferative activity and the possibility that the originally tested natural material was contaminated with trace amounts of an even more active compound.

## Conclusion

In conclusion, the first total synthesis of aromatic sesquiterpine panicein A_2_ (**5**) has been achieved. This synthesis hinges on key steps involving the addition of phenol **16** to carbonate **18** to provide propargyl ether **8** which was then cyclised through a modified Claisen rearrangement to ultimately give the desired cyclic structure of **5**. The correlation of literature values for the isolated natural product and synthetic panicein A_2_ (**5**) confirm the structure of the natural product. Of particular note, when synthetic **5** was tested against a broad range of human cancer cell lines, it was found to exhibit very little activity at 10 μM – this observation is at odds with the earlier report that stated **5** exhibits in vitro cytotoxicity against a number of cell lines (ED_50_ = 5 μg/mL).

## Supporting Information

File 1 Experimental procedures, characterisation data of new compounds, NMR comparison tables of natural and synthetic **5** and NCI testing results sheet.

File 2 ^1^H/^13^C NMR spectra of synthesised compounds.
