# Supplementary material for: Total synthesis of panicein A2
Source: Beilstein J Org Chem. 2015 Oct 26;11:1991–6. doi: 10.3762/bjoc.11.215 (PMC4660959; doi:10.3762/bjoc.11.215)
Supplement: File 1 — Experimental procedures, characterisation data of new compounds, NMR comparison tables of natural and synthetic 5 and NCI testing results sheet. [file Beilstein_J_Org_Chem-11-1991-s001.pdf]

Supporting Information File 1

for

Total synthesis of panicein A_2

Lili Yeung, Lisa I. Pilkington, Melissa M. Cadelis, Brent R. Copp and David Barker*

Address: School of Chemical Sciences, University of Auckland, 23 Symonds Street, Auckland, New Zealand

Email: David Barker - d.barker@auckland.ac.nz

* Corresponding author

Experimental procedures, characterisation data of new compounds, NMR comparison tables of natural and synthetic 5 and NCI testing results sheet

Contents

General experimental details S2

Experimental procedures and compound data S2

Comparison of the NMR data obtained from the isolation of panicein A_2 (**5**) and the NMR data of the synthetic panicein A_2 (**5**) S16

NCI results data sheet S18

References S19

General experimental details:

All reactions were carried out under a nitrogen atmosphere in dry, freshly distilled solvents unless otherwise noted. NMR spectra were recorded on a 400 MHz or 500 MHz spectrometer. Chemical shifts are reported relative to the solvent peak of $CDCl_3$ (δ 7.26 for ^{1}H and δ 77.0 for ^{13}C) or CD_3OD (δ 3.31 for ^{1}H and δ 49.0 for ^{13}C). ^{1}H NMR data is reported as position (δ), relative integral, multiplicity (s, singlet; d, doublet; dd. doublet of doublets; ddq doublet of doublet of quartets; m, multiplet; pd, pentet of doublets, br, broad), coupling constant (*J*, Hz), and the assignment of the atom. ^{13}C NMR data are reported as position (δ) and assignment of the atom. NMR assignments were performed using HSQC and HMBC experiments. High-resolution mass spectrometry (HRMS) was carried out by either chemical ionization (CI) or electrospray ionization (ESI) on a MicroTOF-Q mass spectrometer. Unless noted, chemical reagents were used as purchased.

Experimental procedures and compound data:

2,3,5-Trimethylanisole

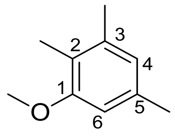

2,3,5-Trimethylphenol **11** (1.52 g, 11.0 mmol) was added to EtOH (30 mL) and H_2O (1.5 mL). KOH (0.73 g, 13.0 mmol) was then added to the mixture and stirred in an ice bath until the solution went clear. Methyl iodide (0.82 mL, 13.2 mmol) was added dropwise to the solution. The solution was stirred for 2 h at rt and then for 12 h at 60 °C. H_2O (50 mL) was added to the reaction mixture and the solution then extracted with Et_2O (1 × 50 mL). The organic extract was washed first with 5% NaOH (20 mL), then H_2O (2 × 20 mL), dried ($MgSO_4$) and the solvent removed in vacuo. The product was purified using silica gel column chromatography in CH_2Cl_2, giving the title compound as a pale yellow oil (1.02 g, 62%).

R_f (CH_2Cl_2) 0.92; **^{1}H NMR ($CDCl_3$, 400 MHz)** δ_H 6.68 (1H, s, H-4), 6.61 (1H, s, H-6), 3.86 (3H, s, OMe), 2.36 (3H, s, 5-CH_3), 2.30 (3H, s, 3-CH_3), 2.18 (3H, s, 2-CH_3); **^{13}C NMR**

($CDCl_3$, 100 MHz) δ_C 157.6 (C-1), 137.6 (C-3), 135.6 (C-5), 123.1 (C-4), 121.9 (C-2), 109.0 (C-6), 55.6 (OMe), 21.4 (5-CH_3), 20.0 (3-CH_3), 11.3 (2-CH_3). The ^{1}H and ^{13}C NMR values are in agreement with literature values.[1]

2,3,6-Trimethyl-4-anisaldehyde (10)

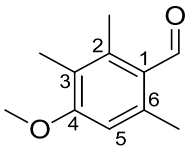

$POCl_3$ (1.49 mL, 16.0 mmol) was added dropwise to stirring dry DMF (1.23 mL, 16.0 mmol) under N_2 in an ice bath. The reaction mixture warmed to 10 °C after the addition, and then the 2,3,5-trimethylanisole (2.08 g, 13.8 mmol) was added at rt. The mixture was then heated to 110 °C for 6 h. The reaction was then cooled to rt and poured on to ice water (50 mL). CH_2Cl_2 (20 mL) and NaOAc (6 g) were added to the mixture and stirred for 1 h. The organic layer was separated and the aqueous layer was extracted with CH_2Cl_2 (3 × 30 mL). The combined organic layers were washed with 2% HCl (20 mL), H_2O (2 × 30 mL) and then dried ($MgSO_4$). The solvent was removed in vacuo, giving the title compound **10** as light yellow crystals (2.02 g, 82%).

R_f (CH_2Cl_2) 0.69; **m.p** 62-66 °C (lit.[1] 63-65 °C); **^{1}H NMR ($CDCl_3$, 400 MHz)** δ_H 10.53 (1H, s, CHO), 6.56 (1H, s, H-5), 3.87 (3H, s, OMe), 2.60 (3H, s, 6-CH_3), 2.53 (3H, s, 2-CH_3), 2.15 (3H, s, 3-CH_3); **^{13}C NMR ($CDCl_3$, 100 MHz)** δ_C 193.0 (CHO), 160.8 (C-4), 141.6 (C-2), 141.3 (C-6), 126.5 (C-1), 124.1 (C-3), 111.0 (C-5), 55.6 (OMe), 21.4 (6-CH_3), 15.9 (2-CH_3), 11.3 (3-CH_3). The ^{1}H and ^{13}C NMR values are in agreement with literature values.[1]

4-(4-Methoxy-2,3,6-trimethylphenyl)but-3-en-2-one (12)

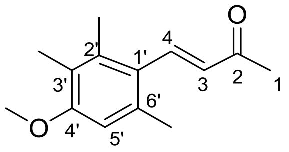

Acetone (15 mL) and H_2O (5 mL) were added to 2,3,6-trimethyl-4-anisaldehyde (**10**, 0.90 g, 5 mmol). 10% aq NaOH (2.8 mL) was added to the stirring mixture in an ice bath over 30 min. The reaction mixture was allowed to stir for 7 h at rt. The reaction was acidified to pH 5 with AcOH and then the solvent removed in vacuo. The residue was dissolved in Et_2O (20 mL), washed with sat. $NaHCO_3$ (20 mL) and H_2O (3 × 20 mL), dried ($MgSO_4$) and the solvent removed in vacuo. The product was purified by silica gel column chromatography (CH_2Cl_2), giving the title compound **12** as pale yellow crystals (0.72 g, 66%).

R_f (CH_2Cl_2) 0.6; **m.p** 59-61 °C (lit.[1] 55-56 °C); **^{1}H NMR ($CDCl_3$, 400 MHz)** δ_H 7.67 (1H, d, *J* = 16.5 Hz, H-4), 6.60 (1H, s, H-5′), 6.22 (1H, d, *J* = 16.5 Hz, H-3), 3.82 (3H, s, OMe), 2.38 (3H, s, 1-CH_3), 2.33 (3H, s, 6′-CH_3), 2.26 (3H, s, 2′-CH_3), 2.15 (3H, s, 3′-CH_3); **^{13}C NMR ($CDCl_3$, 100 MHz)** δ_C 198.6 (C-2), 157.6 (C-4′), 143.2 (C-4), 136.7 (C-2′), 135.0 (C-6′), 132.6 (C-3), 126.8 (C-1′), 123.4 (C-3′), 110.3 (C-5′), 55.6 (OMe), 27.5 (C-1), 21.6 (6′-CH_3), 17.6 (2′-CH_3), 11.9 (3′-CH_3). The ^{1}H and ^{13}C NMR values are in agreement with literature values.[1]

4-(4-Methoxy-2,3,6-trimethylphenyl)but-3-en-2-ol (14)

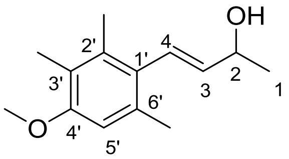

4-(4-Methoxy-2,3,6-trimethylphenyl)but-3-en-2-one **12** (0.431 g, 2.0 mmol) was dissolved in MeOH (1.1 mL), and $NaBH_4$ (0.075 g, 2.0 mmol) was added to the mixture and allowed to

stir in an ice bath for 10 min. Sat. aq NH_4Cl (1 mL) was added to quench the reaction and then extracted with EtOAc (2 × 2 mL). The combined organic extracts were dried ($MgSO_4$) and solvent removed in vacuo to give the title compound **14** as white crystals (0.415 g, 94%).

R_f (4:1 *n*-hexanes:EtOAc) 0.35; **m.p** 65-67 °C; **IR (ATR)** ν_{max} 3312, 2923, 1593, 1456, 1304, 1112 cm^{-1}; **^{1}H NMR ($CDCl_3$, 400 MHz)** δ_H 6.59 (1H, s, H-5′), 6.50 (1H, d, *J* = 16.1 Hz, H-4), 5.63 (1H, dd, *J* = 16.1, 6.4 Hz, H-3), 4.48 (1H, ddq, *J* = 6.4, 6.4, 0.9 Hz, H-2), 3.81 (3H, s, OMe), 2.28 (3H, s, 6′-CH_3), 2.21 (3H, s, 2′-CH_3), 2.14 (3H, s, 3′-CH_3), 1.39 (3H, d, *J* = 6.4 Hz, H-1); **^{13}C NMR ($CDCl_3$, 100 MHz)** δ_C 156.2 (C-4′), 138.8 (C-4), 135.9 (C-2′), 133.8 (C-6′), 129.4 (C-1′), 127.9 (C-3), 122.7 (C-3′), 110.0 (C-5′), 69.5 (C-2), 55.7 (OMe), 23.8 (C-1), 21.4 (6′-CH_3), 17.4 (2′-CH_3), 11.9 (3′-CH_3); ***m/z* (ESI+):** 243 (MNa^+, 100 %) and 226 (20). **(+)-HRMS** $[M+Na]^+$ 243.1353 (calculated for $C_{14}H_{20}O_2Na$, 243.1356).

4-(4-Methoxy-2,3,6-trimethylphenyl)butan-2-ol (15)

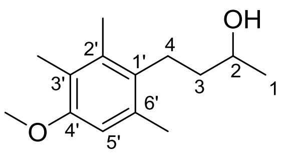

4-(4-Methoxy-2,3,6-trimethylphenyl)but-3-en-2-ol (**14**, 0.415 g, 1.9 mmol) was dissolved in MeOH (14 mL). Pd/C (0.062 g, 0.6 mmol) was added to the mixture and the reaction was purged with H_2. The reaction was stirred under an atmosphere of H_2 at rt for 2 h. TLC was used to determine the reaction had completed and the Pd/C catalyst was removed by filtration using celite, solvent removed in vacuo and the title compound **15** obtained as white crystals (0.349 g, 83%).

R_f (4:1 *n*-hexanes:EtOAc) 0.39; **m.p** 90-92 °C; **IR (ATR)** ν_{max} 3294, 2914, 1594, 1468, 1114 cm^{-1}; **^{1}H NMR ($CDCl_3$, 400 MHz)** δ_H 6.56 (1H, s, H-5′), 3.87 (1H, dddq, *J* = 5.9, 5.9, 5.9, 0.9 Hz, H-2), 3.78 (3H, s, OMe), 2.80–2.72 (1H, m, H_2-4a), 2.65–2.57 (1H, m, H_2-4b), 2.32 (3H, s, 6′-CH_3), 2.23 (3H, s, 2′-CH_3), 2.14 (3H, s, 3′-CH_3), 1.61–1.56 (1H, m, H_2-2), 1.26 (3H, d, *J* = 5.9 Hz, H-1); **^{13}C NMR ($CDCl_3$, 100 MHz)** δ_C 155.4 (C-4′), 136.0 (C-2′), 133.7 (C-6′), 131.0 (C-1′), 123.0 (C-3′), 110.5 (C-5′), 68.5 (C-2), 55.7 (OMe), 39.3 (C-3),

25.9 (C-4), 23.7 (C-1), 20.5 (6′-CH_3), 15.9 (2′-CH_3), 12.1 (3′-CH_3); ***m/z* (ESI+):** 245 (MNa^+, 100 %) and 227 (15). **(+)-HRMS** $[M+Na]^+$ 245.1509 (calculated for $C_{14}H_{22}O_2Na$, 245.1512).

4-(4-Methoxy-2,3,6-trimethylphenyl)butan-2-one (13)

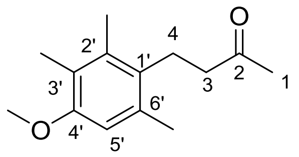

4-(4-Methoxy-2,3,6-trimethylphenyl)butan-2-ol (**15**, 0.349 g, 1.6 mmol) was dissolved in CH_2Cl_2 (10.2 mL). DMP (1.36 g, 3.2 mmol) was added to the mixture which was then stirred for 1 h. TLC was used to determine the reaction had gone to completion. Sat. aq $Na_2S_2O_5$ (80 mL) was used to quench the reaction, followed by $NaHCO_3$ (60 mL) and extracted with CH_2Cl_2 (100 mL). The organic layer was dried ($MgSO_4$) and solvent removed in vacuo to give the title compound **13** as pale yellow crystals (0.315 g, 90%).

R_f (4:1 *n*-hexanes: EtOAc) 0.57; **m.p** 83-90 °C (lit.[1] 86.5-87 °C); **^{1}H NMR ($CDCl_3$, 400 MHz)** δ_H 6.56 (1H, s, H-5′) 3.79 (3H, s, OMe), 2.90–2.86 (2H, m, H_2-4), 2.57–2.53 (2H, m, H_2-3), 2.29 (3H, s, 6′-CH_3), 2.20 (3H, s, 2′-CH_3), 2.17 (3H, s, 1-CH_3), 2.14 (3H, s, 3′-CH_3); **^{13}C NMR ($CDCl_3$, 100 MHz)** δ_C 208.6 (C-2), 155.7 (C-4′), 136.0 (C-2′), 133.8 (C-6′), 129.7 (C-1′), 123.2 (C-3′), 110.5 (C-5′), 55.7 (OMe), 43.6 (C-3), 30.0 (C-1), 23.8 (C-4), 20.5 (6′-CH_3), 15.9 (2′-CH_3), 12.1 (3′-CH_3). The ^{1}H and ^{13}C NMR values are in agreement with literature values.[1]

5-(4-Methoxy-2,3,6-trimethylphenyl)-3-methylpent-1-yn-3-ol (9)

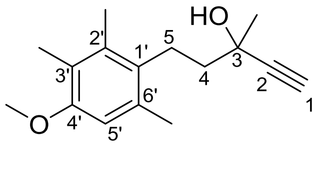

4-(4-Methoxy-2,3,6-trimethylphenyl)butan-2-one (**13**, 91 mg, 4.0 mmol) was dissolved in dry 1:1 THF/Et_2O (2 mL) at −5 °C under an atmosphere of N_2. Ethynylmagnesium bromide

solution (0.5 M, 1.0 mL, 4.8 mmol) was added to the mixture and allowed to stir for 3 h at 0 °C. The reaction was quenched with sat. aq NH_4Cl (5 mL) and the aqueous layer extracted with EtOAc (5 × 10 mL). The organic layer was washed with brine (5 mL) and dried ($MgSO_4$). The solvent was removed in vacuo, giving a yellow crude oil. The pure product was obtained through silica gel column chromatography (9:1 *n*-hexanes/EtOAc) affording title compound **9** as a white solid (93 mg, 95%). **R_f (9:1 *n*-hexanes:EtOAc)** 0.22; **m.p** 62-66 °C (lit.[2] 55.5-57 °C); **^{1}H NMR ($CDCl_3$, 400 MHz)** δ_H 6.57 (1H, s, H-5′), 3.79 (3H, s, OMe), 2.87–2.81 (2H, m, H_2-5), 2.53 (1H, s, H-1), 2.34 (3H, s, 6′-CH_3), 2.24 (3H, s, 2′-CH_3), 2.14 (3H, s, 3′-CH_3), 1.80–1.74 (2H, m, H_2-4), 1.58 (3-CH_3); **^{13}C NMR ($CDCl_3$, 100 MHz)** δ_C 155.5 (C-4′), 136.1 (C-2′), 133.8 (C-6′), 130.2 (C-1′), 122.9 (C-3′), 110.5 (C-5′), 87.6 (C-2), 71.7 (C-1), 68.0 (C-3), 55.6 (OMe), 42.9 (C-4), 29.8 (3-CH_3), 24.8 (C-5), 20.3 (6′-CH_3), 15.7 (2′-CH_3), 12.0 (3′-CH_3). The ^{1}H and ^{13}C NMR values are in agreement with literature values.[2]

Methyl 6-(4-methoxy-2,3,6-trimethylphenyl)-4-((methoxycarbonyl)oxy)-4-methylhex-2-ynoate (19)

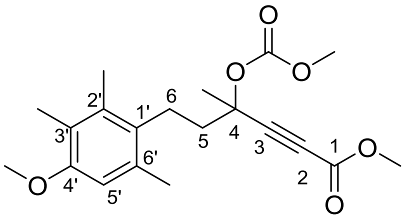

5-(4-Methoxy-2,3,6-trimethylphenyl)-3-methylpent-1-yn-3-ol (**9**, 0.1 g, 0.4 mmol) was dissolved in THF (2 mL). *n*-BuLi (1.6 M, 0.5 mL, 0.8 mmol) was added dropwise to the solution at 0 °C. The reaction was then stirred at this temperature for 30 min. Methyl chloroformate (0.06 mL, 0.8 mmol) was added to the reaction and allowed to stir at rt for 2 h. TLC was used to determine that the reaction had gone to completion. The reaction was quenched with H_2O (5 mL) and extracted with CH_2Cl_2 (5 mL). The aqueous layer was further extracted with Et_2O (5 mL) and dried ($MgSO_4$), solvent removed in vacuo to give a yellow crude oil. The compound was purified using silica gel chromatography (9:1 *n*-hexane/EtOAc), yielding the title compound **19** a colourless oil (0.063 g, 43%). **^{1}H NMR ($CDCl_3$, 400 MHz)** δ_H 6.56 (1H, s, H-5′), 3.81 (3H, s, OMe), 3.80 (3H, s, OMe), 3.79 (3H, s, OMe), 2.75 (2H, pd, *J* = 5.2, 13.6 Hz, H_2-6), 2.32 (3H, s, 6′-CH_3), 2.23 (3H, s, 2′-CH_3), 2.14

(3H, s, 3′-CH_3), 2.12–2.06 (1H, m, H_2-5a), 2.01-1.93 (1H, m, H_2-5b), 1.83 (3-CH_3); **^{13}C NMR ($CDCl_3$, 100 MHz)** δ_C 155.7 (C-4′), 153.7 (C=O), 153.6 (C=O), 136.1 (C-2′), 133.9 (C-6′), 129.2 (C-1′), 123.1 (C-3′), 110.5 (C-5′), 86.3 (C-3), 77.4 (C-2), 76.0 (C-4), 55.7 (OMe), 54.9 (OMe), 53.0 (OMe), 40.5 (C-5), 25.7 (4-CH_3), 24.2 (C-6), 20.3 (6′-CH_3), 15.7 (2′-CH_3), 12.1 (3′-CH_3). In a separate fraction, **18** (0.021 g, 17%) as a pale yellow oil (see below for full characterisation data).

5-(4-Methoxy-2,3,6-trimethylphenyl)-3-methylpent-1-yn-3-yl methyl carbonate (18)

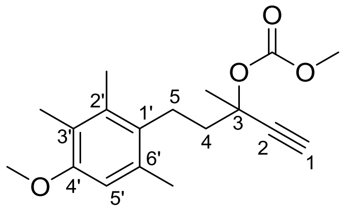

5-(4-Methoxy-2,3,6-trimethylphenyl)-3-methylpent-1-yn-3-ol (**9**, 0.1 g, 0.4 mmol) was dissolved in THF (2 mL). *n*-BuLi (1.6 M, 0.5 mL, 0.8 mmol) was added dropwise to the solution stirring at 0 °C. The reaction was then stirred at this temperature for 30 min. Methyl chloroformate (0.46 mL, 0.6 mmol) was added to the reaction and allowed to stir at rt for 2 h. TLC was used to determine that the reaction had gone to completion. The reaction was quenched with H_2O (5 mL) and extracted with CH_2Cl_2 (5 mL). The aqueous layer was further extracted with Et_2O (5 mL) and dried ($MgSO_4$), solvent removed in vacuo to give a yellow crude oil. The compound was purified using silica gel chromatography (9:1 *n*-hexane/EtOAc), yielding the title compound **18** a pale yellow oil (0.062 g, 51%). **R_f (9:1 *n*-hexanes:EtOAc)** 0.53; **^{1}H NMR ($CDCl_3$, 400 MHz)** δ_H 6.58 (1H, s, H-5′), 3.80 (3H, s, OMe), 3.80 (3H, s, OMe), 2.90–2.81 (2H, m, H_2-5), 2.69 (1H, s, H-1), 2.35 (3H, s, 6′-CH_3), 2.26 (3H, s, 2′-CH_3), 2.15 (3H, s, 3′-CH_3), 2.14–2.06 (1H, m, H_2-4a), 1.96-1.88 (1H, m, H_2-4b), 1.82 (3-CH_3); **^{13}C NMR ($CDCl_3$, 100 MHz)** δ_C 155.6 (C-4′), 153.8 (C=O), 136.1 (C-2′), 133.9 (C-6′), 129.6 (C-1′), 123.0 (C-3′), 110.5 (C-5′), 83.1 (C-2), 76.8 (C-3), 74.2 (C-1), 55.6 (OMe), 54.5 (OMe), 40.9 (C-4), 26.3 (3-CH_3), 24.3 (C-5), 20.3 (6′-CH_3), 15.7 (2′-CH_3), 12.0 (3′-CH_3). The ^{1}H and ^{13}C NMR values are in agreement with literature values.[2]

4-Hydroxyphenyl acetate (16)

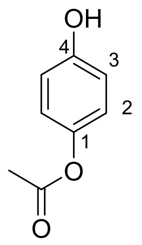

Hydroquinone (1.0 g, 9.1 mmol) was dissolved in AcOH (2 mL) under N_2. Acetic anhydride (0.43 mL, 4.5 mmol) was added dropwise to the mixture over 30 min. The mixture was allowed to stir at 110 °C for 2 h. The solvent was removed in vacuo and the crude mixture was dissolved in toluene. Unreacted starting material was removed by filtration and the solvent was removed in vacuo affording title compound **16** as a pale brown-yellow solid (0.55 g, 40%). **R_f (2:1 *n*-hexanes: EtOAc)** 0.77; **m.p** 50-52 °C (lit.[3] 60-62 °C); **^{1}H NMR ($CDCl_3$, 400 MHz)** δ_H 6.88 (2H, d, *J*= 8.8 Hz, H-2), 6.71 (2H, d, *J*= 8.9 Hz, H-3), 2.28 (3H, s, $COCH_3$); **^{13}C NMR ($CDCl_3$, 100 MHz)** δ_C 170.9 (**C**OCH_3), 153.7 (C-4), 144.0 (C-1), 122.4 (C-2), 116.2 (C-3), 21.2 (CO**C**H_3). The ^{1}H and ^{13}C NMR values are in agreement with literature values.[3]

4-((5-(4-Methoxy-2,3,6-trimethylphenyl)-3-methylpent-1-yn-3-yl)oxy)phenyl acetate (8) and 4-((5-(4-methoxy-2,3,6-trimethylphenyl)-3-methylpent-1-yn-3-yl)oxy)phenol (20)

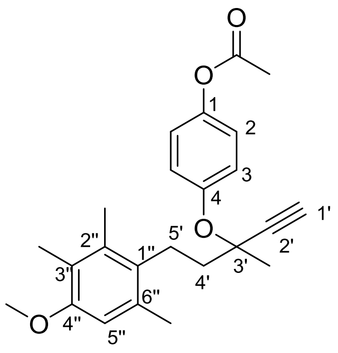

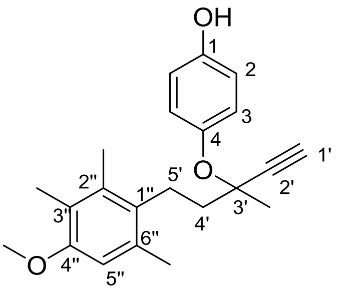

The phenol **16** (98 mg, 0.64 mmol) was dissolved in MeCN (0.7 mL). Anhydrous copper(II) chloride (0.01 mg, 0.064 mmol) and DBU (0.12 mL, 0.83 mmol) was added to the mixture at −20 °C and stirred for 15 min. The carbonate **18** (195 mg, 0.64 mmol) in MeCN (1.8 mL) was then added dropwise and stirred overnight at 0 °C. The reaction was quenched with H_2O

(5 mL) and extracted with Et_2O (3 × 5 mL). The organic layer was dried ($MgSO_4$) and the solvent removed in vacuo. A brown crude oil purified using silica gel column chromatography (4:1 *n*-hexanes/EtOAc) yielded title compound **6** as a pale yellow oil (42 mg, 17 %) and title compound **20** as a colourless oil (45 mg, 21%).

4-((5-(4-Methoxy-2,3,6-trimethylphenyl)-3-methylpent-1-yn-3-yl)oxy)phenyl acetate (8)

R_f (4:1 *n*-hexanes:EtOAc) 0.51; **IR (ATR)** ν_{max} 3282, 2936, 2153, 1761, 1596, 1500, 1466, 1369, 1212, 1171, 1118, 1091, 1013 cm^{-1}; **^{1}H NMR (CD_3OD, 400 MHz)** δ_H 7.23 (2H, d, *J* = 9.1 Hz, H-2), 7.00 (2H, d, *J* = 9.1 Hz, H-3), 6.58 (1H, s, H-5″), 3.75 (3H, s, OMe), 3.14 (1H, s, H-1′), 2.99–2.83 (2H, m, H_2-5′), 2.28 (3H, s, 6″-CH_3), 2.25 (3H, s, $OCOCH_3$), 2.21 (3H, s, 2″-CH_3), 2.09 (3H, s, 3″-CH_3), 1.98–1.85 (2H, m, H_2-4′), 1.62 (3′-CH_3); **^{13}C NMR (CD_3OD, 100 MHz)** δ_C 171.4 (O**C**OCH_3), 156.8 (C-4″), 154.6 (C-4), 147.6 (C-1), 136.7 (C-2″), 134.8 (C-6″), 131.2 (C-1″), 123.6 (C-3″), 123.3 (C-2), 123.0 (C-3) 111.5 (C-5″), 85.8 (C-2′), 77.3 (C-1′), 77.0 (C-3′), 56.0 (OMe), 43.3 (C-4′), 27.3 (3′-CH_3), 25.5 (C-5′), 20.9 (OCO**C**H_3), 20.4 (6″-CH_3), 15.8 (2″-CH_3), 12.1 (3″-CH_3); ***m/z* (ESI+):** 403 (MNa^+, 100 %), 341 (90), 265 (50) and 168 (80); **(+)-HRMS** $[M+Na]^+$ 403.1869 (calculated for $C_{24}H_{28}O_4Na$, 403.1880).

4-((5-(4-Methoxy-2,3,6-trimethylphenyl)-3-methylpent-1-yn-3-yl)oxy)phenol (20)

R_f (4:1 *n*-hexanes:EtOAc) 0.30; **IR (ATR)** ν_{max} 3394, 3273, 2984, 2112, 1596, 1505, 1449, 1369, 1279, 1214, 1174, 1116 cm^{-1}; **^{1}H NMR ($CDCl_3$, 400 MHz)** δ_H 7.10 (2H, d, *J* = 8.9 Hz, H-3), 6.74 (2H, d, *J* = 8.9 Hz, H-2), 6.59 (1H, s, H-5″), 4.91 (1H, br s, OH), 3.81 (3H, s, OMe), 3.02–2.85 (2H, m, H_2-5′), 2.63 (1H, s, H-1′), 2.35 (3H, s, 6″-CH_3), 2.26 (3H, s, 2″-CH_3), 2.16 (3H, s, 3″-CH_3), 2.04–1.86 (2H, m, H_2-4′), 1.61 (3′-CH_3); **^{13}C NMR ($CDCl_3$, 100 MHz)** δ_C 155.5 (C-4″), 151.7 (C-4), 149.2 (C-1), 136.3 (C-2″), 134.0 (C-6″), 130.4 (C-1″), 123.8 (C-3), 123.0 (C-3″), 115.5 (C-2) 110.6 (C-5″), 85.3 (C-2′), 76.1 (C-3′), 75.3 (C-1′), 55.7 (OMe), 42.1 (C-4′), 27.0 (3′-CH_3), 24.8 (C-5′), 20.4 (6″-CH_3), 15.8 (2″-CH_3), 12.1 (3″-CH_3); ***m/z* (ESI+):** 361 (MNa^+, 100 %) and 217 (15); **(+)-HRMS $[M+Na]^+$** 361.1774 (calculated for $C_{22}H_{26}O_3Na$, 361.1778).

4-((5-(4-Methoxy-2,3,6-trimethylphenyl)-3-methylpent-1-yn-3-yl)oxy)phenyl acetate (8)

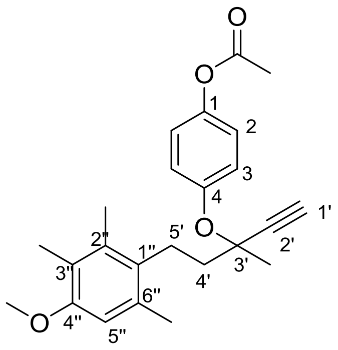

Phenol **20** (19 mg, 0.056 mmol) was dissolved in pyridine (0.05 mL) and stirred under N_2. Acetic anhydride (0.05 mL, 0.56 mmol) was added to the mixture, dropwise and was then allowed to stir at rt for 1.5 h. The reaction was quenched with H_2O (5 mL) and extracted with CH_2Cl_2 (5 mL). The organic layer was then washed with NH_4Cl (5 mL), water (5 mL) and dried ($MgSO_4$). The solvent was removed in vacuo giving the crude product as a colourless oil. The compound was purified using silica gel chromatography (4:1 *n*-hexanes/EtOAc), yielding the title compound **8** a colourless oil (0.014 g, 66%). Data as reported above.

O-Acetyl panicein A_2 (21)

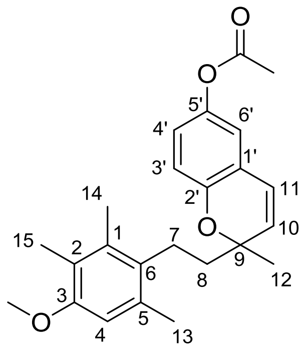

The phenyl acetate **8** (42 mg, 0.1 mmol) was degassed in a solution of toluene (8 mL) and heated in an atmosphere of N_2 under reflux for 2 days. The solvent was removed in vacuo and the crude product was purified using silica gel column chromatography (4:1 *n*-hexanes/EtOAc) to give the title compound **21** in a 1:1 mixture of starting material and product**.** The product was used immediately without further purification.

R_f (4:1 *n*-hexanes:EtOAc) 0.54; **^{1}H NMR ($CDCl_3$, 400 MHz)** δ_H 6.82 (1H, d, *J* = 2.8 Hz, H-4'), 6.82 (1H, s, H-6'), 6.74 (1H, d, *J* = 2.8 Hz, H-3'), 6.55 (1H, s, H-4), 6.36 (1H, d, *J* = 9.8 Hz, H-11), 5.68 (1H, d, *J* = 9.8, H-10), 3.81 (3H, s, OMe), 2.82–2.69 (2H, m, H_2-7), 2.29 (3H, s, $OCOCH_3$), 2.27 (3H, s, H_3-13), 2.20 (3H, s, H_3-14), 2.14 (3H, s, H_3-15), 1.85–1.73 (2H, m, H_2-8), 1.65 (H_3-12); **^{13}C NMR ($CDCl_3$, 100 MHz)** δ_C 169.9 (O**C**OCH$_3$), 155.4 (C-3), 151.0 (C-2'), 144.2 (C-5'), 136.0 (C-1), 133.7 (C-5), 130.3 (C-10), 130.3 (C-6), 123.0 (C-2), 123.0 (C-1'), 122.8 (C-11), 121.9 (C-4'), 119.2 (C-3'), 116.7 (C-6'), 110.5 (C-4), 79.0 (C-9), 55.7 (OMe), 41.0 (C-8), 26.5 (C-12), 24.1 (C-7), 21.2 (OCO**C**H$_3$), 20.4 (C-13), 15.7 (C-14), 12.1 (C-15).

tert-Butyl(4-((5-(4-methoxy-2,3,6-trimethylphenyl)-3-methylpent-1-yn-3-yl)oxy)phenoxy)dimethylsilane (22)

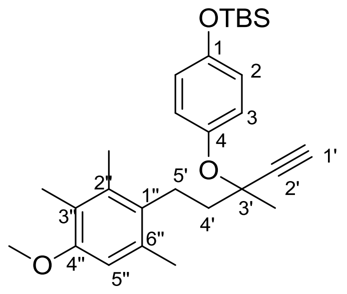

To a solution of phenol **20** (22 mg, 0.065 mmol) in CH_2Cl_2 (2 mL) with DIPEA (0.045 mL, 0.26 mmol) stirred under an atmosphere of N_2 at 0 °C was added TBSCl (0.28 mL, 0.16 mmol). The solution was allowed to warm to room temperature and was stirred for 24 h. Sat. aq $NaHCO_3$ (2 mL) was added and the layers separated. The aqueous layer was extracted with ether (3 × 3 mL) and the combined organic extracts were dried ($MgSO_4$). The solvent was removed in vacuo giving the crude product as colourless oil. The compound was purified using silica gel chromatography (4:1 *n*-hexanes/EtOAc), yielding the title compound **22** a colourless oil (5.3 mg, 18%).

^{1}H NMR ($CDCl_3$, 400 MHz) δ_H 7.09 (2H, d, *J* = 8.0 Hz, H-2), 6.74 (2H, d, *J* = 8.0 Hz, H-3), 6.56 (1H, s, H-5″), 3.79 (3H, s, OMe), 3.00–2.83 (2H, m, H_2-5′), 2.61 (1H, s, H-1′), 2.32 (3H, s, 6″-CH_3), 2.24 (3H, s, 2″-CH_3), 2.14 (3H, s, 3″-CH_3), 2.01–1.85 (2H, m, H_2-4′), 1.59 (3′-CH_3), 0.98 (9H, s, Si$(CH_3)_2$C(C*H*$_3$)$_3$), 0.18 (9H, s, Si(C*H*$_3$)$_2$C$(CH_3)_3$); **^{13}C NMR ($CDCl_3$, 100 MHz)** δ_C 155.4 (C-4″), 151.6 (C-4), 149.7 (C-1), 136.3 (C-2″), 134.0 (C-6″), 130.4 (C-1″), 123.2 (C-3″), 123.0 (C-2), 120.2 (C-3) 110.5 (C-5″), 85.4 (C-2′), 75.8 (C-3′), 75.1 (C-1′), 55.7 (OMe), 42.1 (C-4′), 29.8 (3′-CH_3), 27.0 (C-5′), 25.8 (Si$(CH_3)_2$C(*C*H$_3$)$_3$), 24.7 (Si$(CH_3)_2$*C*$(CH_3)_3$) 20.4 (6″-CH_3), 15.8 (2″-CH_3), 12.1 (3″-CH_3), -4.3 (Si(*C*H$_3$)$_2$C$(CH_3)_3$).

_O_-TBS panicein A_2 (23)

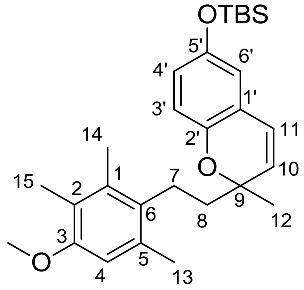

The phenyl silane **22** (5.3 mg, 0.012 mmol) was degassed in a solution of toluene (4 mL) and heated in an atmosphere of N_2 under reflux for 2 days. The solvent was removed in vacuo and the crude product was purified using silica gel column chromatography (4:1 *n*-hexanes/EtOAc) to give the title compound **23** in a 1:2 mixture of starting material and product**.**

^{1}H NMR ($CDCl_3$, 400 MHz) δ_H 6.69 (1H, d, *J* = 8.0 Hz, H-4'), 6.60 (1H, dd, *J* = 8.0 and 2.4 Hz, H-6'), 6.53 (1H, s, H-4), 6.48 (1H, d, *J* = 2.4 Hz, H-3'), 6.33 (1H, d, *J* = 9.8 Hz, H-11), 5.65 (1H, d, *J* = 9.8, H-10), 3.77 (3H, s, OMe), 2.79–2.70 (2H, m, H_2-7), 2.26 (3H, s, H_3-13), 2.17 (3H, s, H_3-14), 2.11 (3H, s, H_3-15), 1.78–1.72 (2H, m, H_2-8), 1.45 (H_3-12), 0.98 (9H, s, $Si(CH_3)_2C(C\mathit{H}_3)_3$), 0.17 (9H, s, $Si(C\mathit{H}_3)_2C(CH_3)_3$); **^{13}C NMR ($CDCl_3$, 100 MHz)** δ_C 155.4 (C-3), 151.4 (C-2'), 145.8 (C-5'), 135.9 (C-1), 133.6 (C-5), 130.2 (C-10), 130.2 (C-6), 123.2 (C-2), 123.2 (C-1'), 122.9 (C-11), 120.2 (C-3'), 117.5 (C-4'), 116.6 (C-6'), 110.4 (C-4), 78.0 (C-9), 55.6 (OMe), 40.3 (C-8), 26.9 (C-12), 25.9 ($Si(CH_3)_2\mathit{C}(CH_3)_3$), 25.7 ($Si(CH_3)_2C(\mathit{C}H_3)_3$), 23.9 (C-7), 20.3 (C-13), 15.6 (C-14), 12.0 (C-15), -4.5 ($Si(\mathit{C}H_3)_2C(CH_3)_3$).

Panicein A_2 (5)

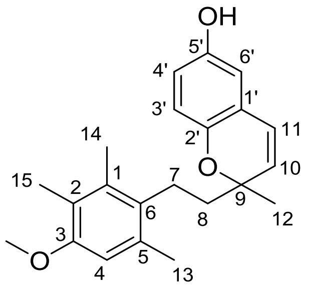

A mixture of chromenol acetate **21** and phenyl acetate **8** (0.019 g, 0.05 mmol) was dissolved in MeOH (1 mL), followed by the addition of aq KOH (1 M, 0.05 mL). The mixture was allowed to stir for 2.5 h, neutralised with AcOH (0.05 mL) and extracted with H_2O (5 mL) and EtOAc (3 × 5 mL). The combined organic layers were dried ($MgSO_4$) and the solvent removed in vacuo to give the crude product which was purified by silica gel column chromatography (9:1 *n*-hexanes/EtOAc), to give the title compound **5** as a pale yellow oil (13.5 mg, 80%).

R_f (4:1 *n*-hexanes:EtOAc) 0.32; **IR (ATR)** v_{max} 3321, 2934, 2252, 1650, 1532, 1453, 1362, 1221, 1116 cm^{-1}; **^{1}H NMR (CD_3OD, 500 MHz)** δ_H 6.61 (1H, d, *J* = 8.6 Hz, H-3'), 6.54 (1H, dd, *J* = 8.6, 2.8 Hz, H-4'), 6.54 (1H, s, H-4), 6.47 (1H, d, *J* = 2.8 Hz, H-6'), 6.34 (1H, d, *J* = 9.8 Hz, H-11), 5.71 (1H, d, *J* = 9.8 Hz, H-10), 3.73 (3H, s, OMe), 2.78–2.66 (2H, m, H_2-7), 2.22 (3H, s, H-13), 2.14 (3H, s, H-14), 2.06 (3H, s, H-15), 1.72–1.65 (2H, m, H_2-8), 1.41 (1H, s, H-12); **^{13}C NMR (CD_3OD, 125 MHz)** δ_C 156.6 (C-3), 152.1 (C-2'), 147.5 (C-5'), 136.5 (C-1), 134.6 (C-5), 131.9 (C-6), 131.6 (C-10), 124.2 (C-11), 123.5 (C-2), 123.2 (C-1') 117.5 (C-3'), 116.4 (C-4'), 113.8 (C-6'), 111.4 (C-4), 79.1 (C-9), 56.0 (OMe), 41.4 (C-8), 26.2 (C-12), 24.9 (C-7), 20.4 (C-13), 15.8 (C-14), 12.0 (C-15); ***m/z* (ESI+):** 361 (MNa^+, 100 %) **(+)-HRMS** $[M+Na]^+$ 361.1774 (calculated for $C_{22}H_{26}O_3Na$, 361.1778). The ^{1}H and ^{13}C NMR values are in agreement with literature values.[4]

Comparison of the NMR data obtained from the isolation of panicein A_2 (5) and the NMR data of the synthetic panicein A_2 (5).

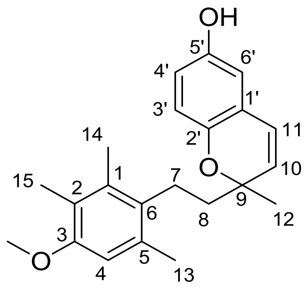

Table 1: ^{1}H NMR Values (CD_3OD) observed for synthetic (**5**) versus literature.

**Atom no.**	**Isolated panicein A_2 (5) (400 MHz, CD_3OD)[4]**	**Synthetic panicein A_2 (5) (500 MHz, CD_3OD)**
H-4	6.53 (1H, s)	6.54 (1H, s)
H-7	2.71 (2H, m)	2.78–2.66 (2H, m)
H-8	1.68 (2H, m)	1.72–1.65 (2H, m)
H-10	5.71 (1H, d, *J* = 9.8 Hz)	5.71 (1H, d, *J* = 9.8 Hz)
H-11	6.35 (1H, d, *J* = 9.8 Hz)	6.34 (1H, d, *J* = 9.8 Hz)
H-12	1.40 (3H, s)	1.41 (3H, s)
H-13	2.22 (3H, s)	2.22 (3H, s)
H-14	2.13 (3H, s)	2.14 (3H, s)
H-15	2.06 (3H, s)	2.06 (3H, s)
OCH_3	3.72 (3H, s)	3.73 (3H, s)
H-3'	6.60 (1H, d, *J* = 8.6 Hz)	6.61 (1H, d, *J* = 8.6 Hz)
H-4'	6.54 (1H, dd, *J* = 8.6, 2.8 Hz)	6.54 (1H, dd, *J* = 8.6, 2.8 Hz)
H-6'	6.46 (1H, d, *J* = 2.8 Hz)	6.47 (1H, d, *J* = 2.8 Hz)

Table 2. ^{13}C NMR Values (CD_3OD) observed for synthetic (**5**) versus literature.

**Atom no.**	**Isolated panicein A$_2$ (5) (100 MHz, CD$_3$OD)**[4]	**Synthetic panicein A$_2$ (5) (125 MHz, CD$_3$OD)**
C-1	135.4	136.5
C-2[a]	122.6	123.5
C-3	155.3	156.6
C-4	110.7	111.4
C-5	133.6	134.6
C-6	130.9	131.9
C-7	25.2	24.9
C-8	41.5	41.4
C-9	78.7	79.1
C-10	130.6	131.6
C-11	123.3	124.2
C-12	26.5	26.2
C-13	20.8	20.4
C-14	16.2	15.8
C-15	12.5	12.0
OCH_3	55.8	56.0
C-1'[a]	122.3	123.2
C-2'	150.9	152.1
C-3'	116.7	117.5
C-4'	115.6	116.4
C-5'	146.4	147.5
C-6'	113.0	113.8

a) Notates interchangeable signals

NCI results data sheet of synthetic panicein A_2 (5)

Developmental Therapeutics Program One Dose Mean Graph	NSC: D-783527 / 1	Conc: 1.00E-5 Molar	Test Date: Mar 09, 2015
	Experiment ID: 1503OS44		Report Date: Mar 26, 2015

Panel/Cell Line	Growth Percent
Leukemia	
CCRF-CEM	85.68
HL-60(TB)	93.68
K-562	89.45
MOLT-4	85.71
RPMI-8226	84.00
SR	85.89
Non-Small Cell Lung Cancer	
A549/ATCC	86.66
EKVX	94.37
HOP-62	99.30
HOP-92	96.65
NCI-H226	96.59
NCI-H23	93.90
NCI-H322M	106.38
NCI-H460	101.55
NCI-H522	85.39
Colon Cancer	
COLO 205	102.15
HCC-2998	104.39
HCT-116	96.60
HCT-15	100.65
HT29	102.96
KM12	100.46
SW-620	103.45
CNS Cancer	
SF-268	96.88
SF-295	96.23
SF-539	98.40
SNB-19	111.14
SNB-75	78.38
U251	94.16
Melanoma	
LOX IMVI	99.73
MALME-3M	121.50
M14	90.92
MDA-MB-435	102.76
SK-MEL-2	95.31
SK-MEL-5	99.35
Ovarian Cancer	
IGROV1	95.61
OVCAR-3	113.08
OVCAR-4	87.47
OVCAR-5	101.05
OVCAR-8	89.37
NCI/ADR-RES	98.87
SK-OV-3	108.85
Renal Cancer	
786-0	104.57
A498	99.04
ACHN	92.26
CAKI-1	91.76
RXF 393	110.29
SN12C	89.30
TK-10	113.73
UO-31	98.70
Prostate Cancer	
PC-3	74.11
DU-145	107.78
Breast Cancer	
MCF7	96.13
MDA-MB-231/ATCC	87.10
HS 578T	79.38
BT-549	108.22
T-47D	57.34
MDA-MB-468	105.72
Mean	96.32
Delta	38.98
Range	64.16

Mean Growth Percent - Growth Percent

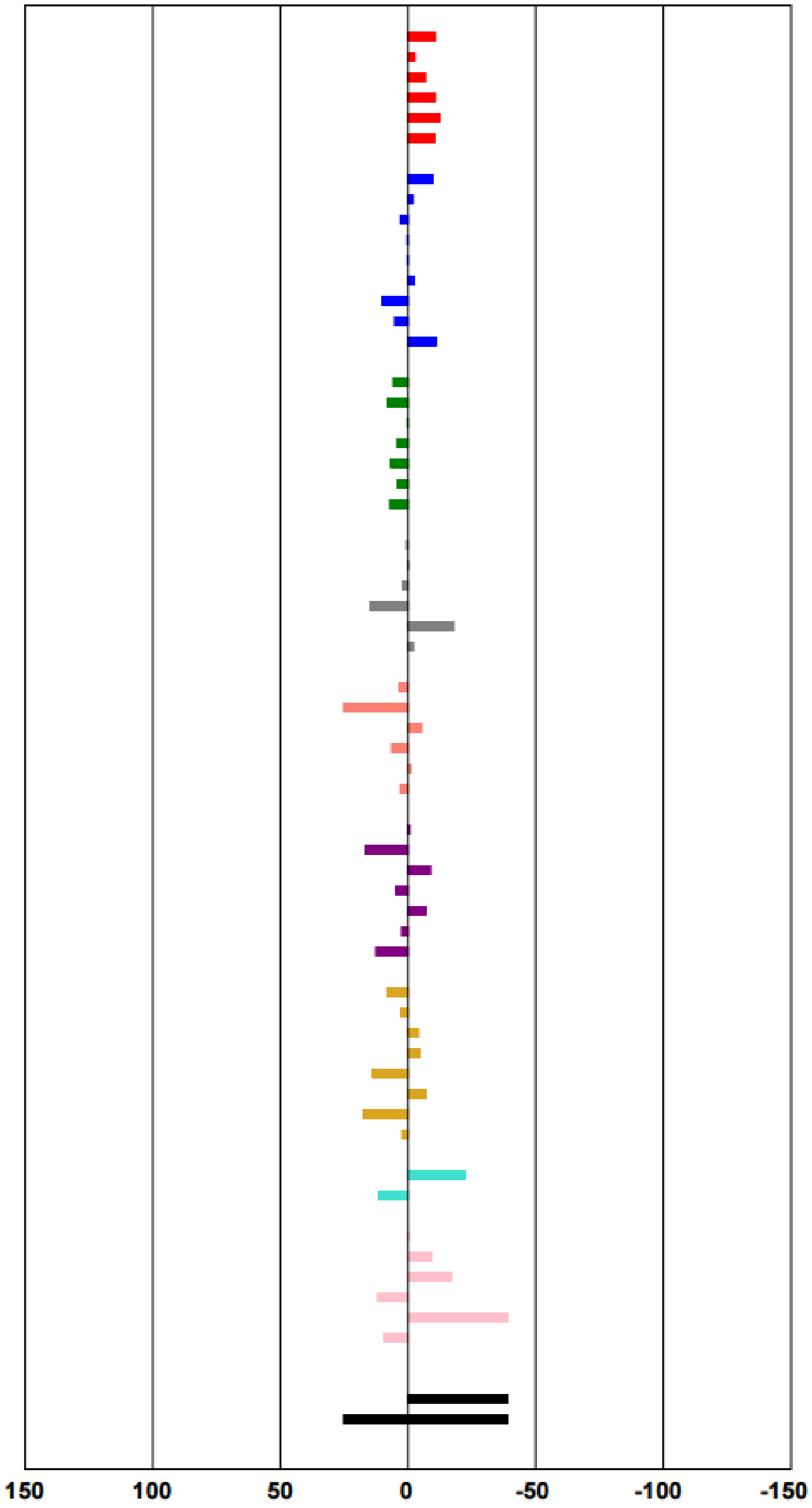

References:

(1) Samokhvalov, G. I.; Zakharova, N. I.; Sokolova, N. N.; Filippova, T. M.; Raigorodskaya, O. I.; Murav'ev, V. V.; Tuguzova, A. M.; Khristoforov, V. L. *Pharm. Chem. J.* **1994**, *28*, 343–348.

(2) Davis, C. J.; Hurst, T. E.; Jacob, A. M.; Moody, C. J. *J. Org. Chem.* **2005**, *70*, 4414–4422.

(3) Kim, B.-T.; Kim, K.-H.; Kim, K.-S.; Lee, Y.-S.; Min, Y.-K.; Park, N.-K. Mono carbonylation of benzene diols. WO2003033449 A1, April 24, 2003.

(4) Zubía, E.; J. Ortega, M.; Luis Carballo, J.; Salvá, J. *Tetrahedron* **1994**, *50*, 8153–8160.
