# Supplementary material for: Total synthesis of panicein A2
Source: Beilstein J Org Chem. 2015 Oct 26;11:1991–6. doi: 10.3762/bjoc.11.215 (PMC4660959; doi:10.3762/bjoc.11.215)
Supplement: File 2 — 1H/13C NMR spectra of synthesised compounds. [file Beilstein_J_Org_Chem-11-1991-s002.pdf]

Supporting Information File 2

for

Total synthesis of panicein A_2

Lili Yeung, Lisa I. Pilkington, Melissa M. Cadelis, Brent R. Copp and David Barker*

Address: School of Chemical Sciences, University of Auckland, 23 Symonds Street, Auckland, New Zealand

Email: David Barker - d.barker@auckland.ac.nz

* Corresponding author

^{1}H/^{13}C NMR spectra of synthesised compounds

Contents:

^{1}H NMR and ^{13}C NMR spectra for 2,3,5-trimethylanisole	S3,S4
^{1}H NMR and ^{13}C NMR spectra for **10**	S5,S6
^{1}H NMR and ^{13}C NMR spectra for **12**	S7,S8
^{1}H NMR and ^{13}C NMR spectra for **14**	S9,S10
^{1}H NMR and ^{13}C NMR spectra for **15**	S11,S12
^{1}H NMR and ^{13}C NMR spectra for **13**	S13,S14
^{1}H NMR and ^{13}C NMR spectra for **9**	S15,S16
^{1}H NMR and ^{13}C NMR spectra for **19**	S17,S18
^{1}H NMR and ^{13}C NMR spectra for **18**	S19,S20

^{1}H NMR and ^{13}C NMR spectra for **16** S21,S22

^{1}H NMR and ^{13}C NMR spectra for **8** S23,S24

^{1}H NMR and ^{13}C NMR spectra for **20** S25,S26

^{1}H NMR and ^{13}C NMR spectra for **20** and **21** S27,S28

^{1}H NMR and ^{13}C NMR spectra for **22** S29,S30

^{1}H NMR and ^{13}C NMR spectra for **22** and **23** S31,S32

^{1}H NMR and ^{13}C NMR spectra for **5** S33,S34

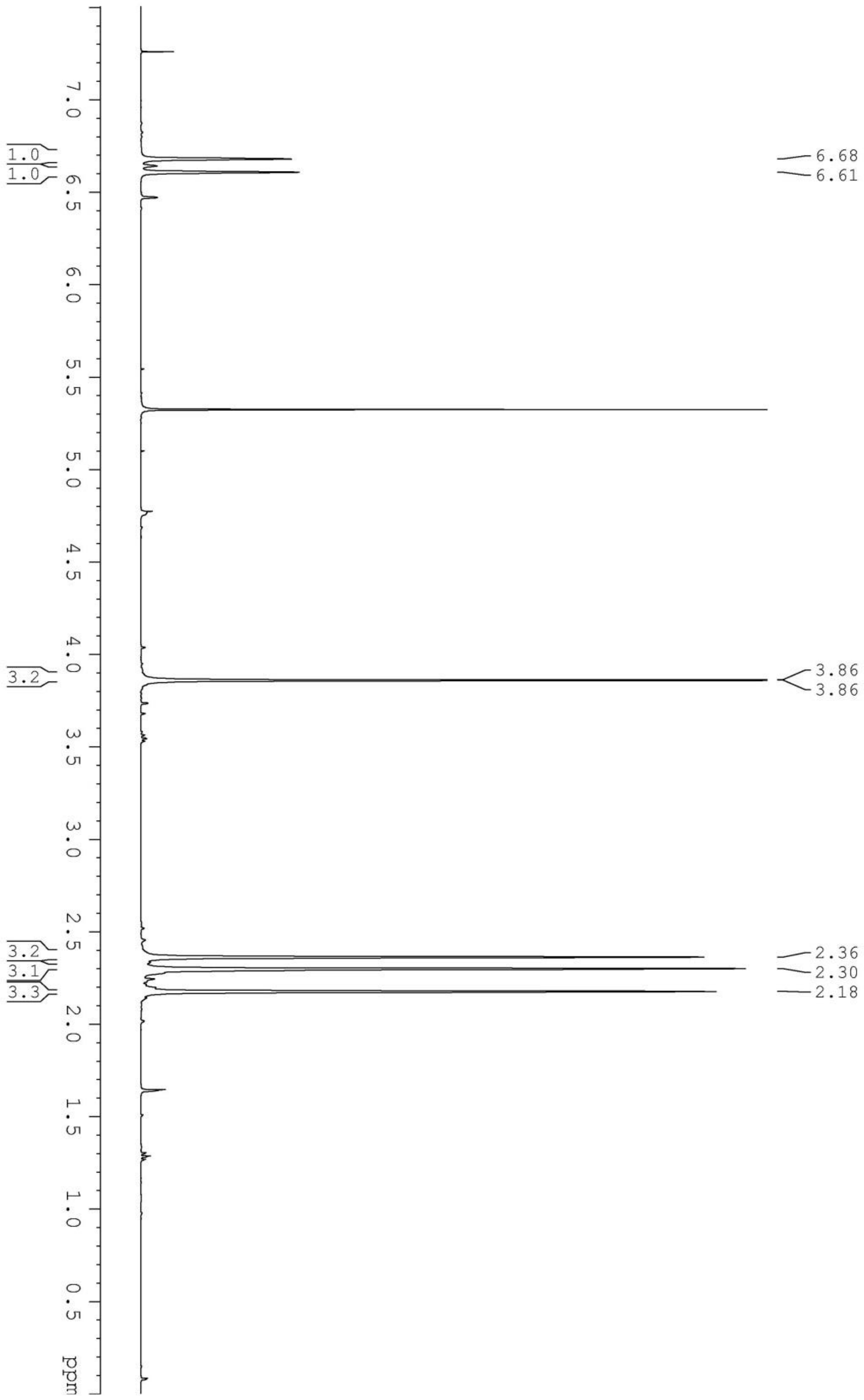

Figure 1: ^{1}H NMR of 2,3,5-trimethylanisole ($CDCl_3$, 400 MHz).

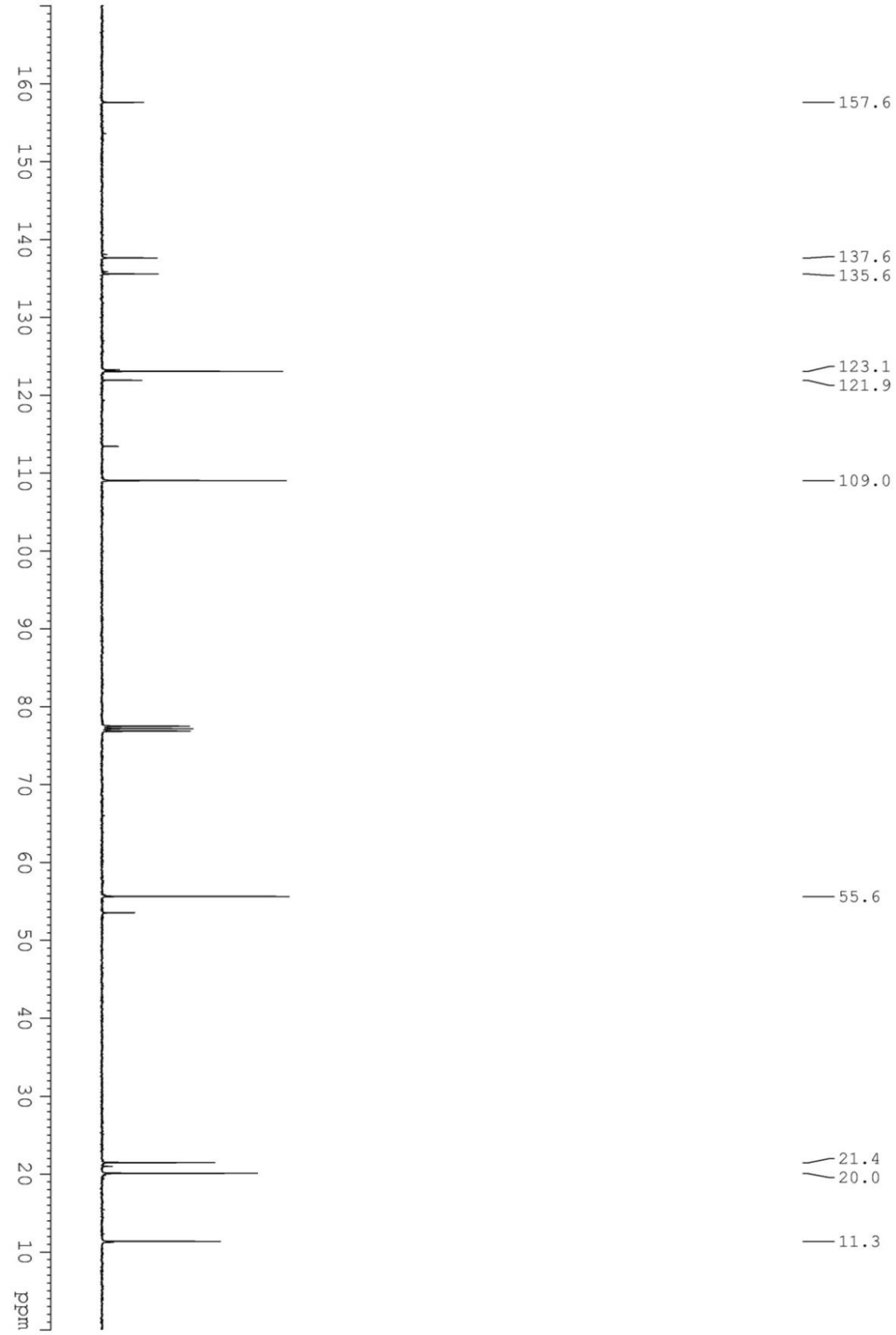

Figure 2: ^{13}C NMR of 2,3,5-trimethylanisole ($CDCl_3$, 100 MHz).

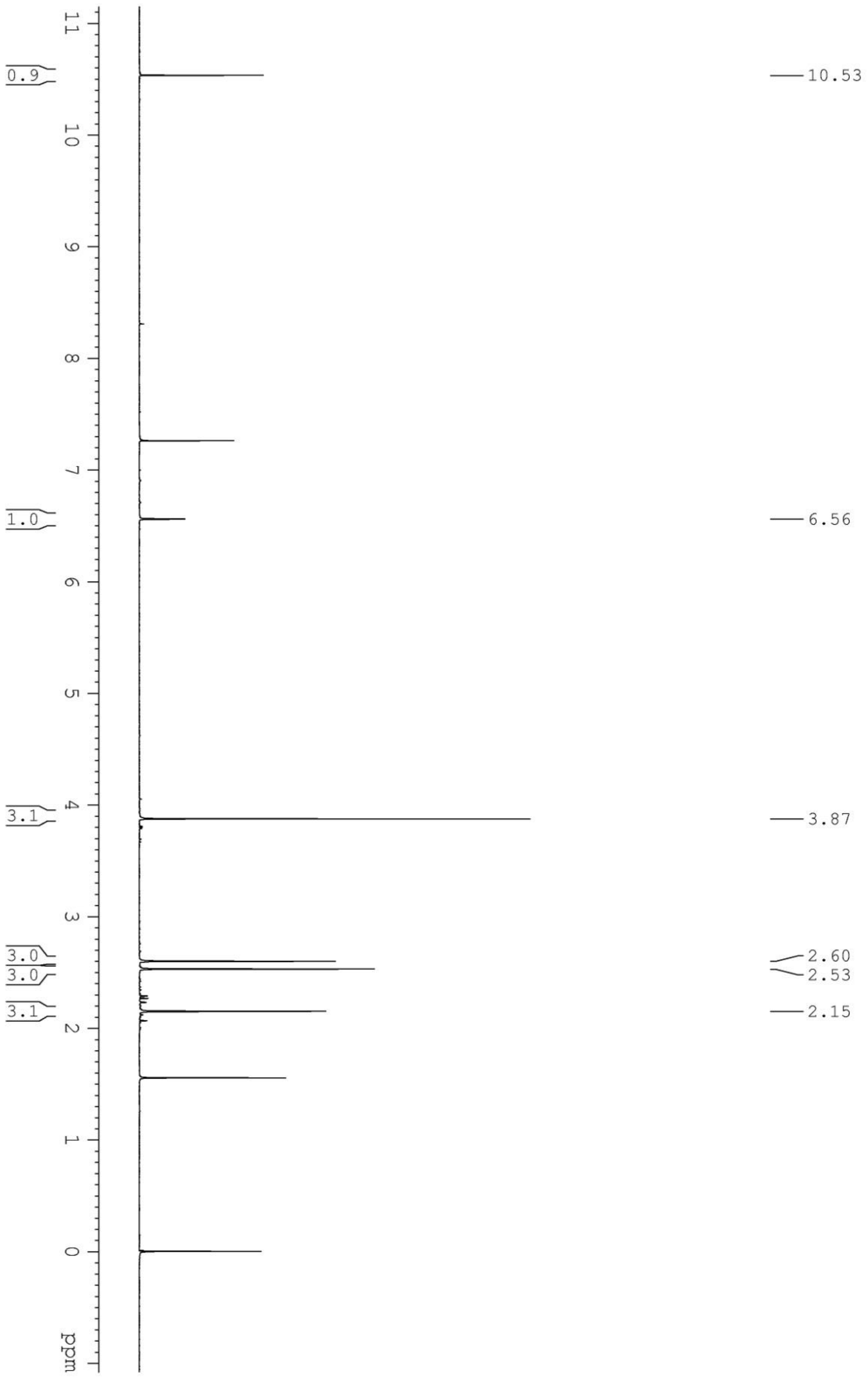

Figure 3: ^{1}H NMR of **10** ($CDCl_3$, 400 MHz).

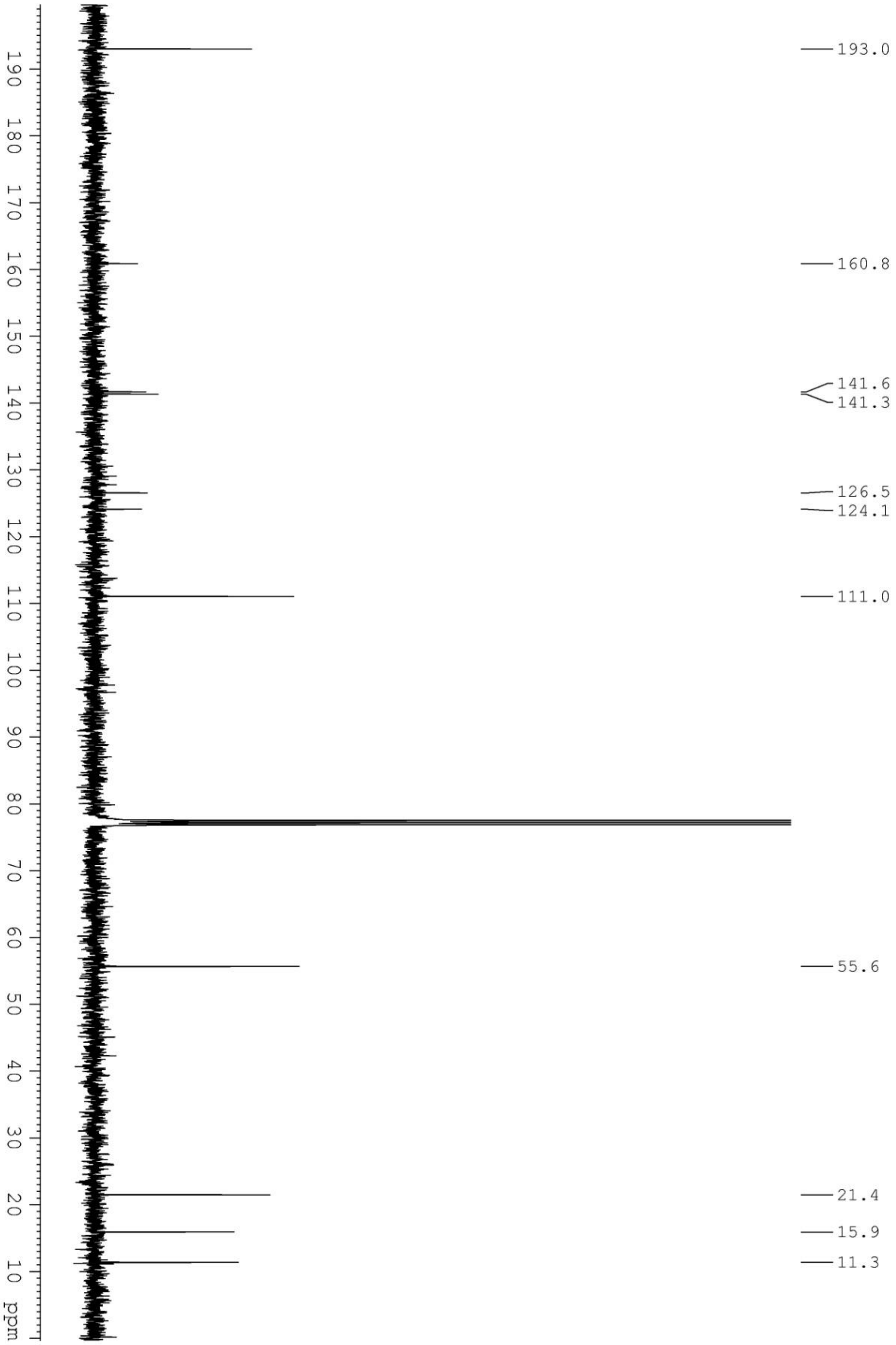

Figure 4: ^{13}C NMR of **10** ($CDCl_3$, 100 MHz).

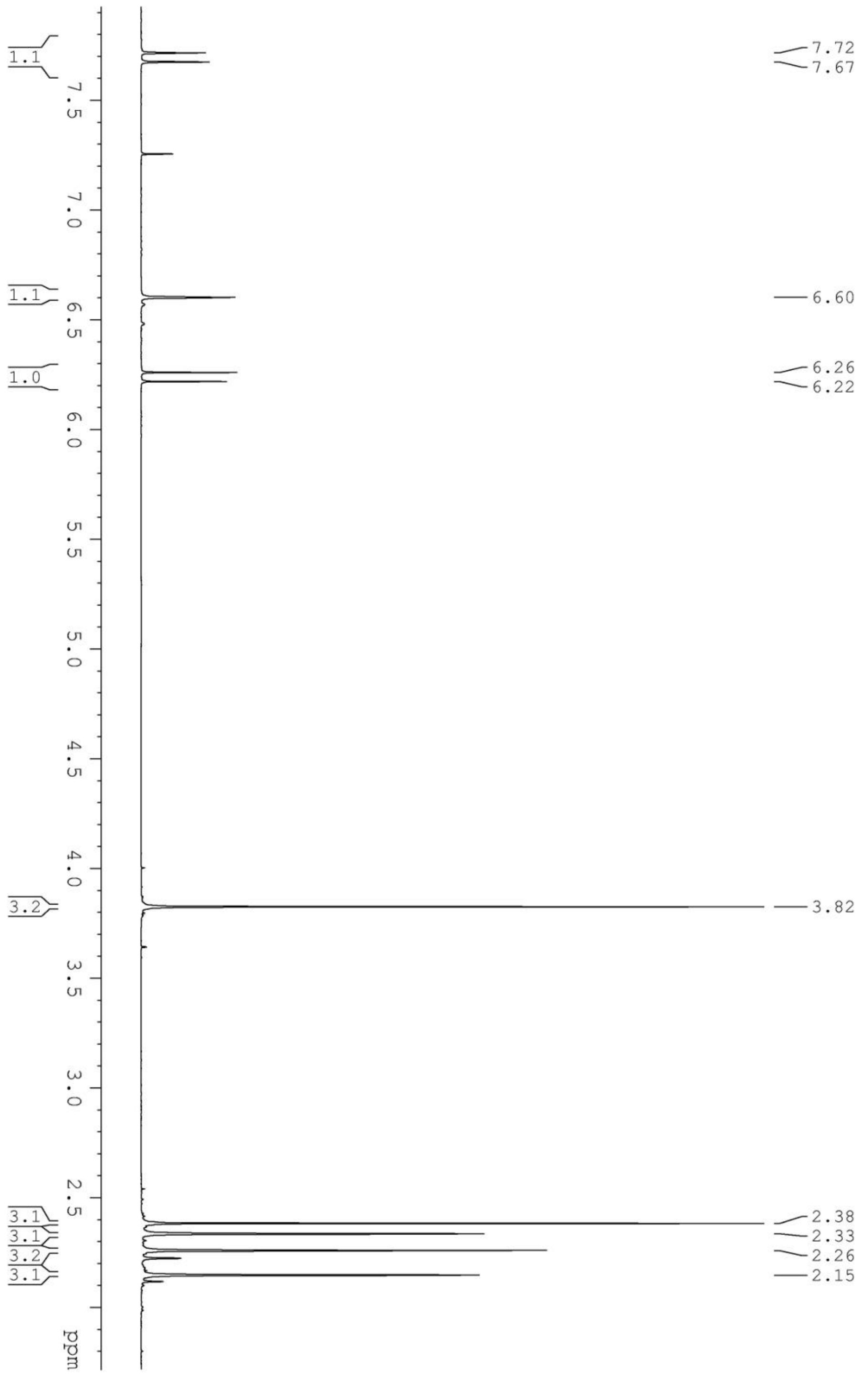

Figure 5: ^{1}H NMR of **12** ($CDCl_3$, 400 MHz).

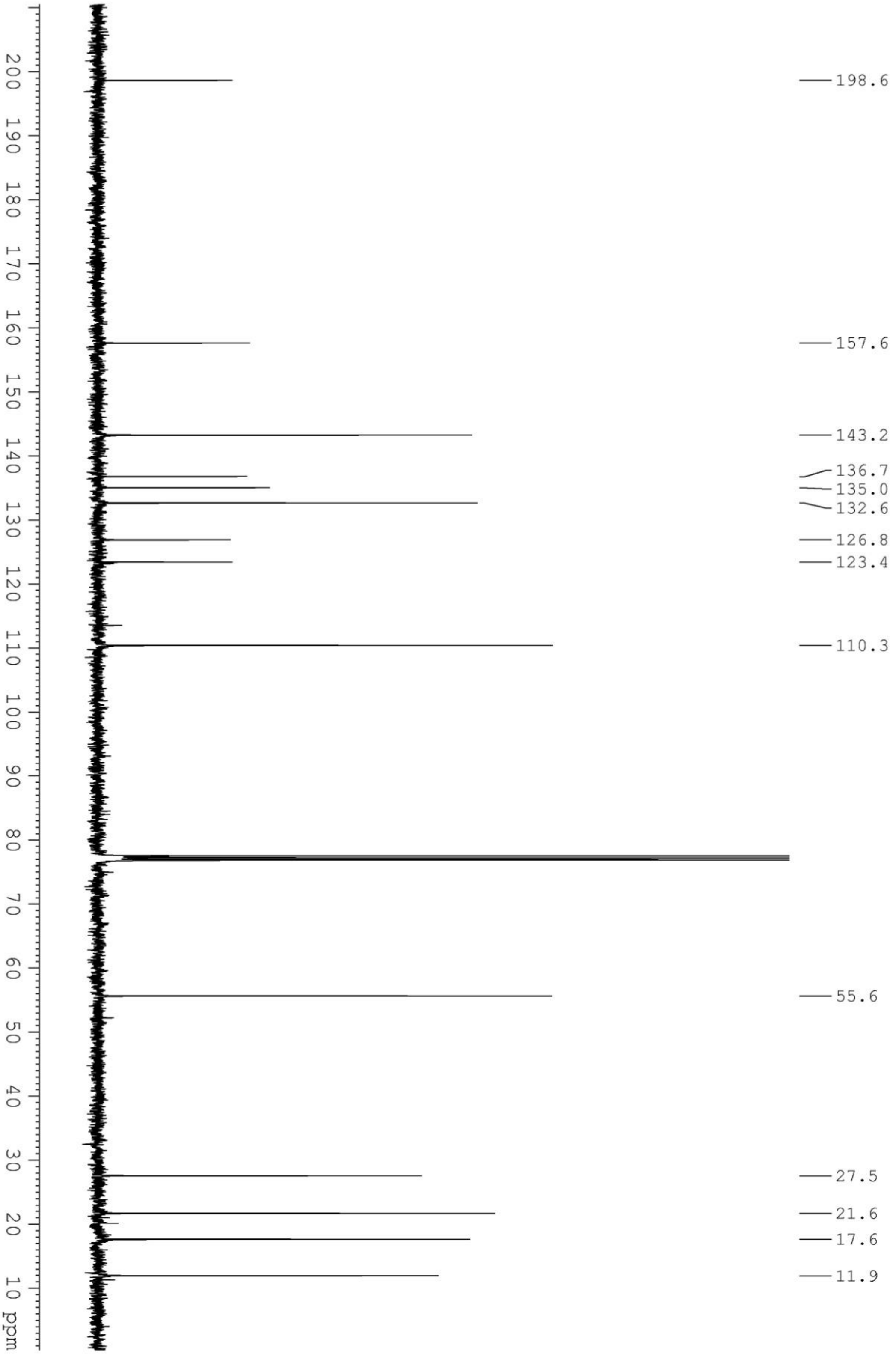

Figure 6: ^{13}C NMR of **12** ($CDCl_3$, 400 MHz).

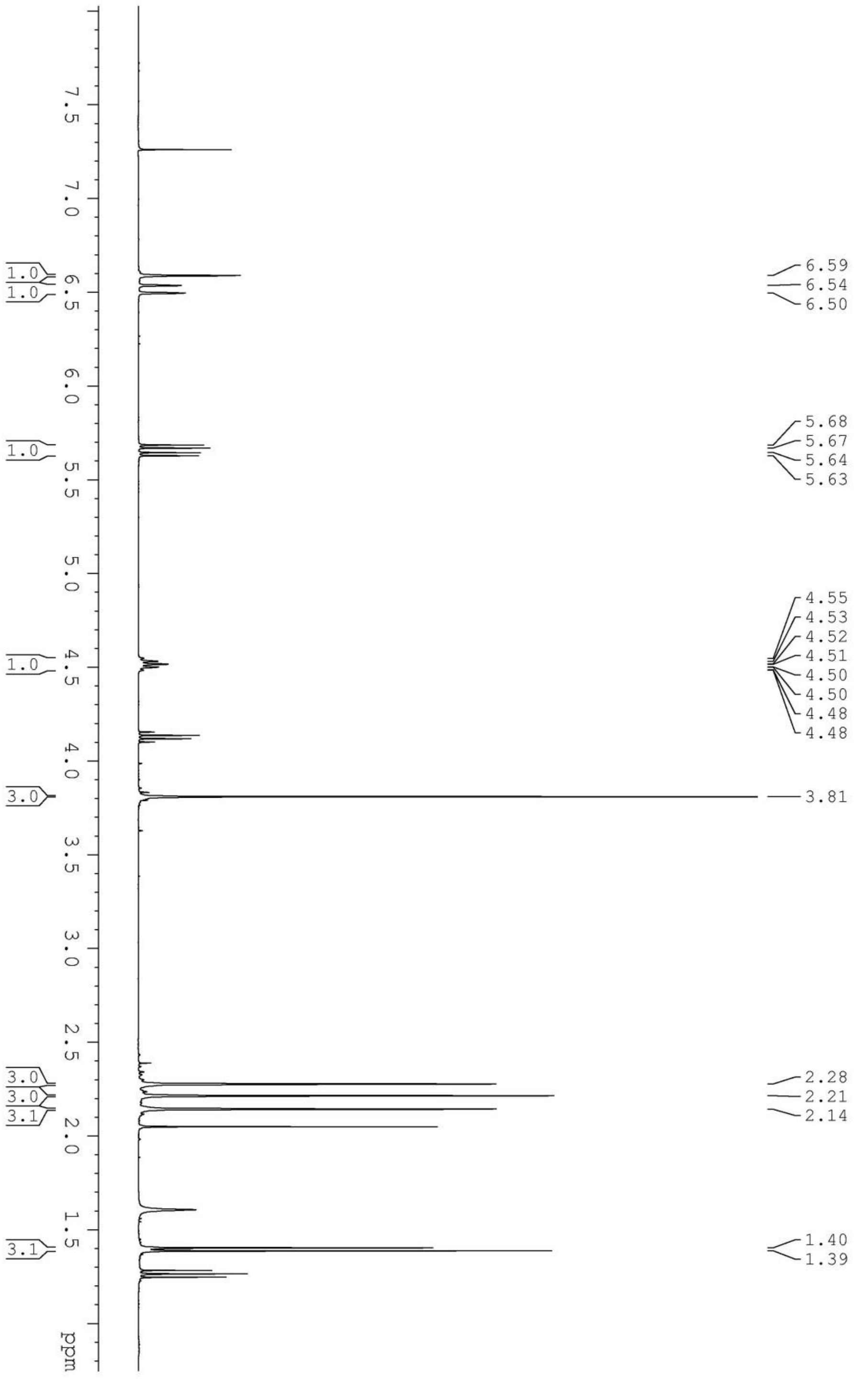

Figure 7: ^{1}H NMR of **14** ($CDCl_3$, 400 MHz).

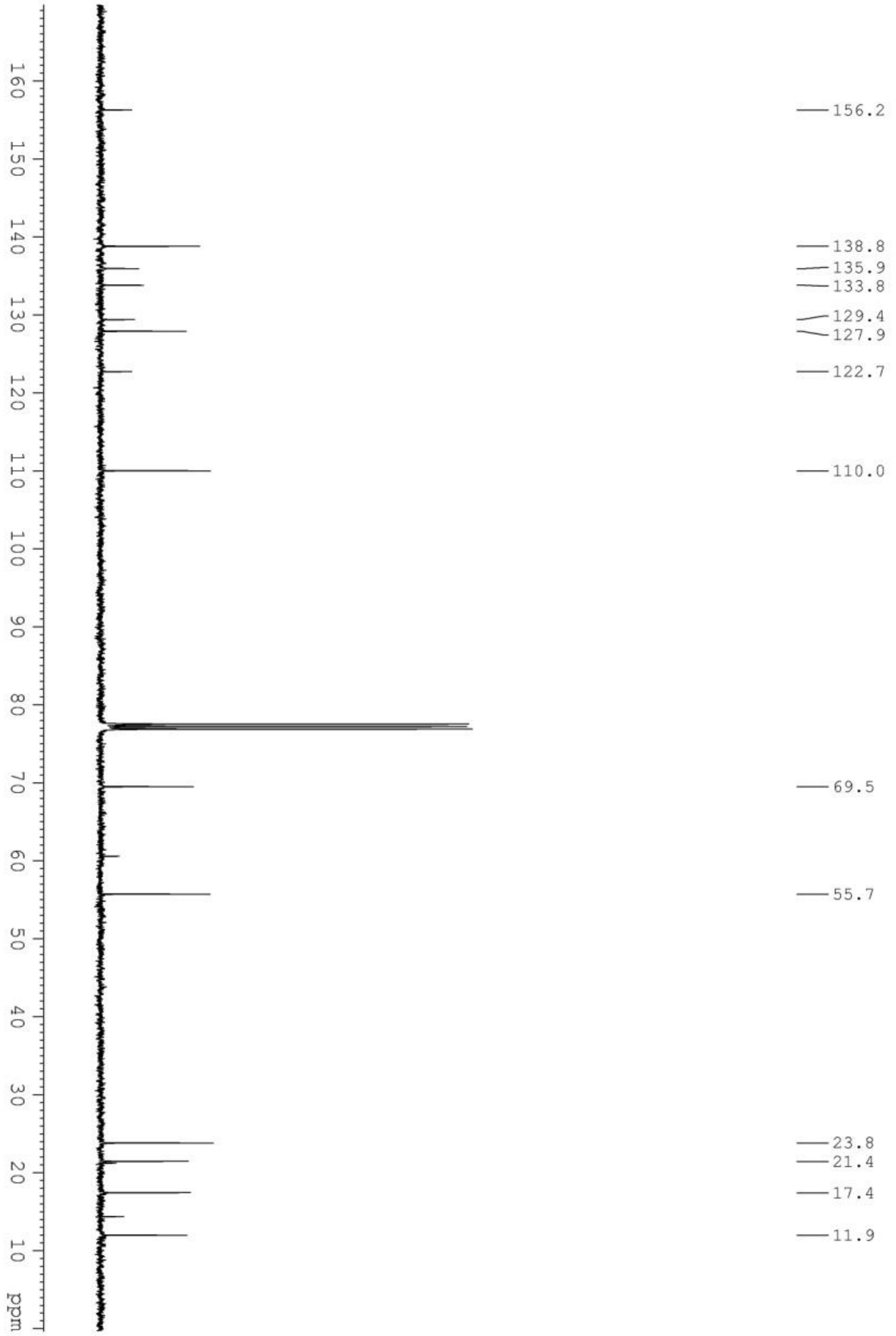

Figure 8: ^{13}C NMR of **14** ($CDCl_3$, 100 MHz).

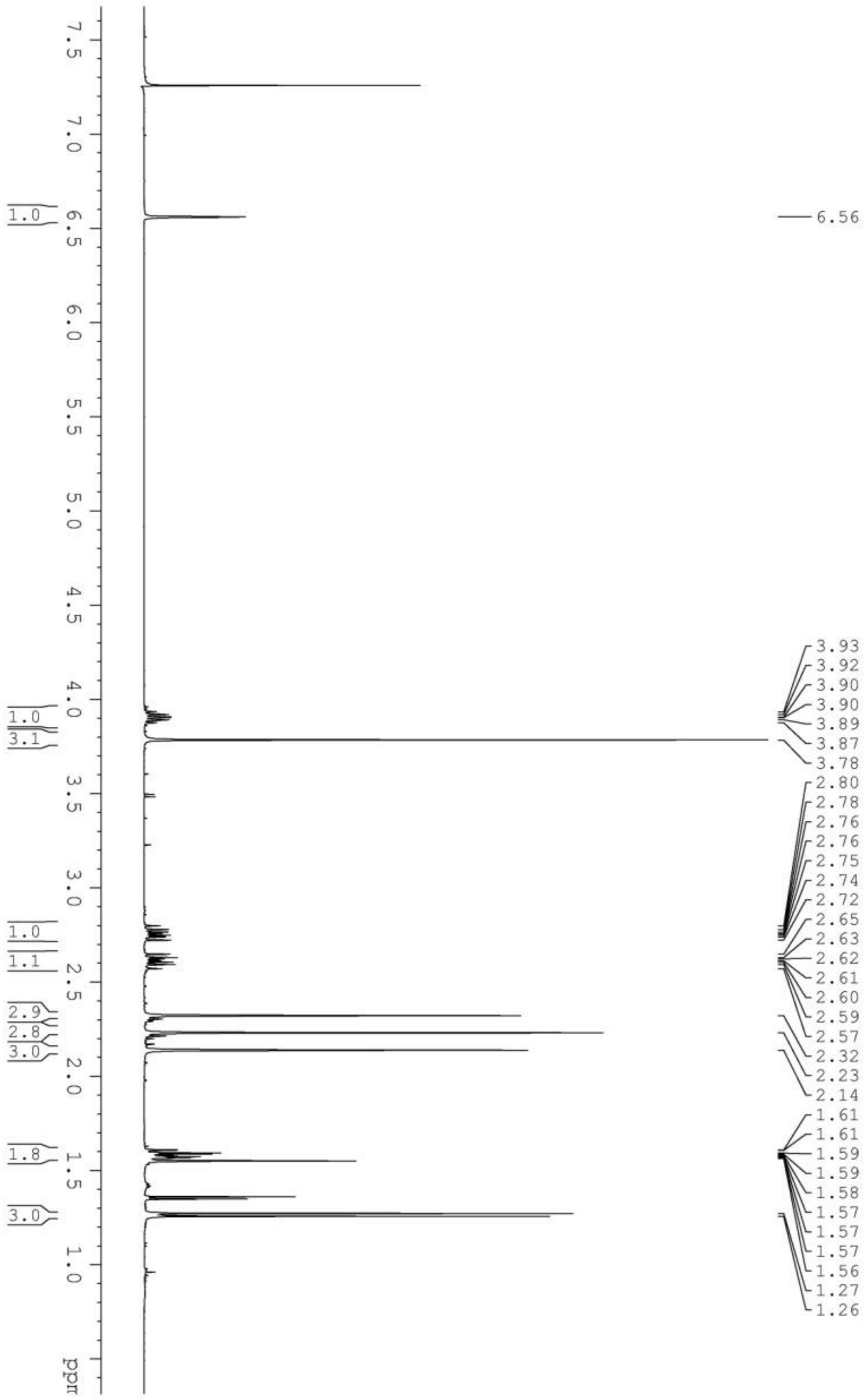

Figure 9: ^{1}H NMR of **15** ($CDCl_3$, 400 MHz).

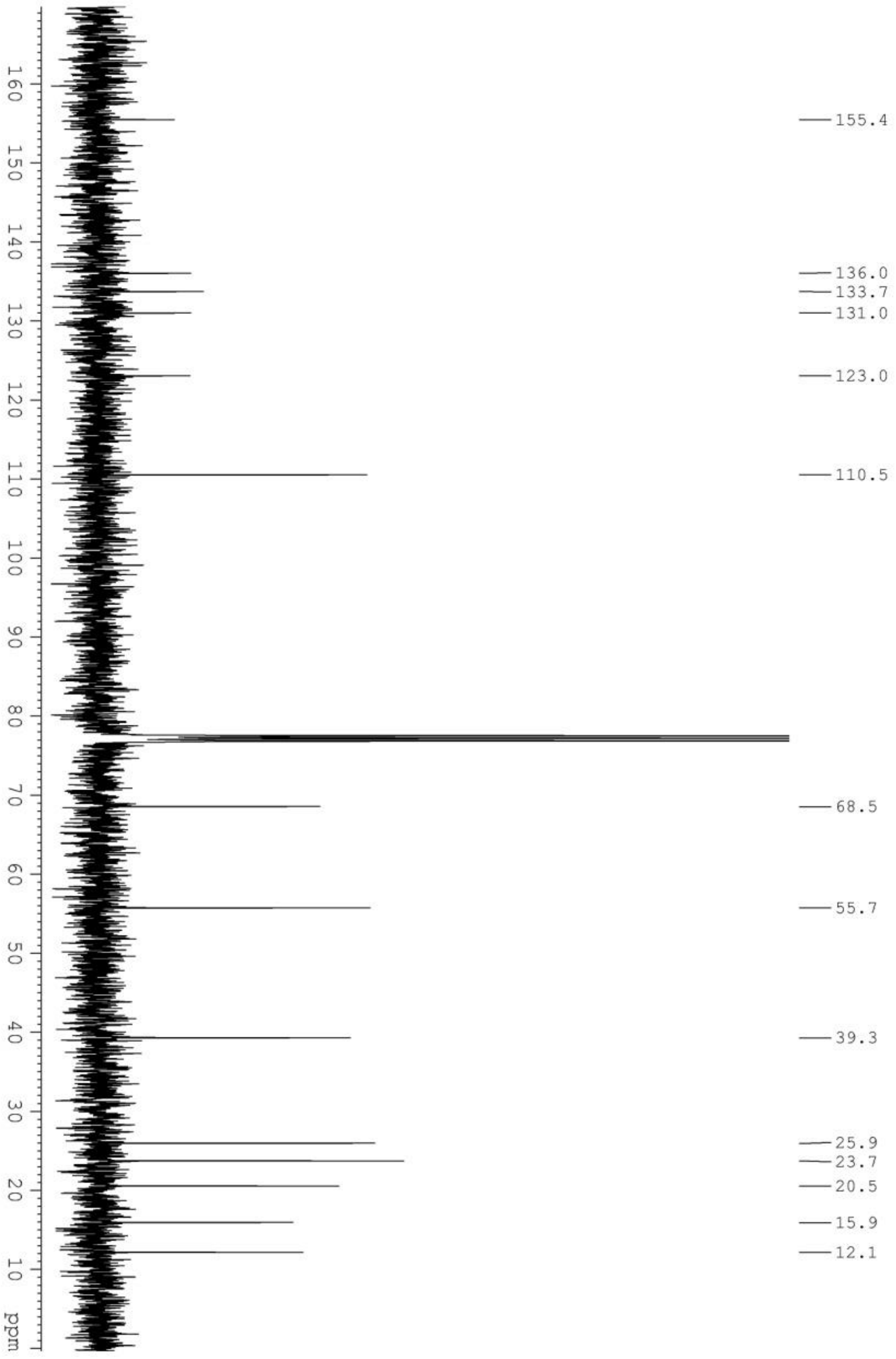

Figure 10: ^{13}C NMR of **15** ($CDCl_3$, 100 MHz).

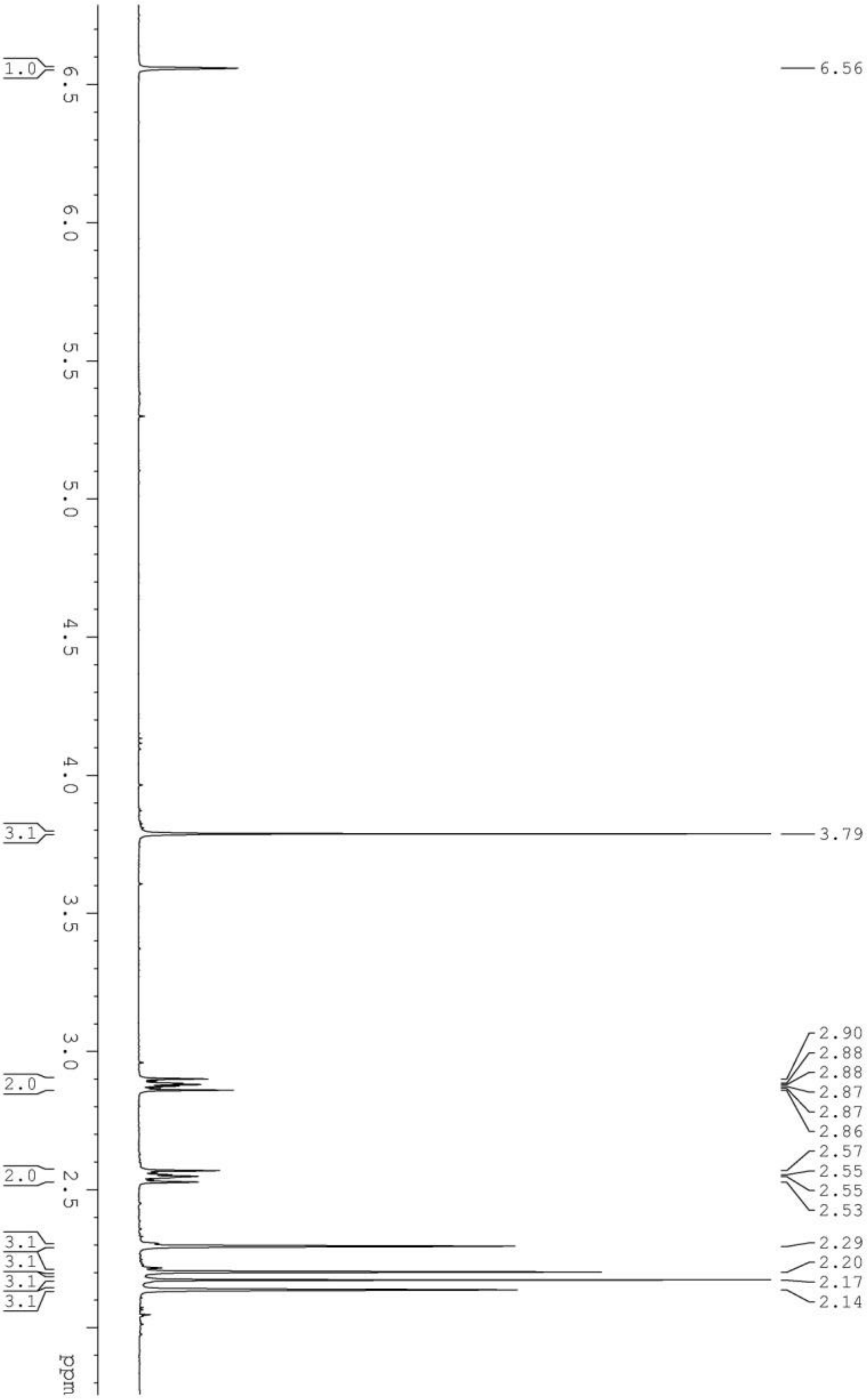

Figure 11: ^{1}H NMR of **13** ($CDCl_3$, 400 MHz).

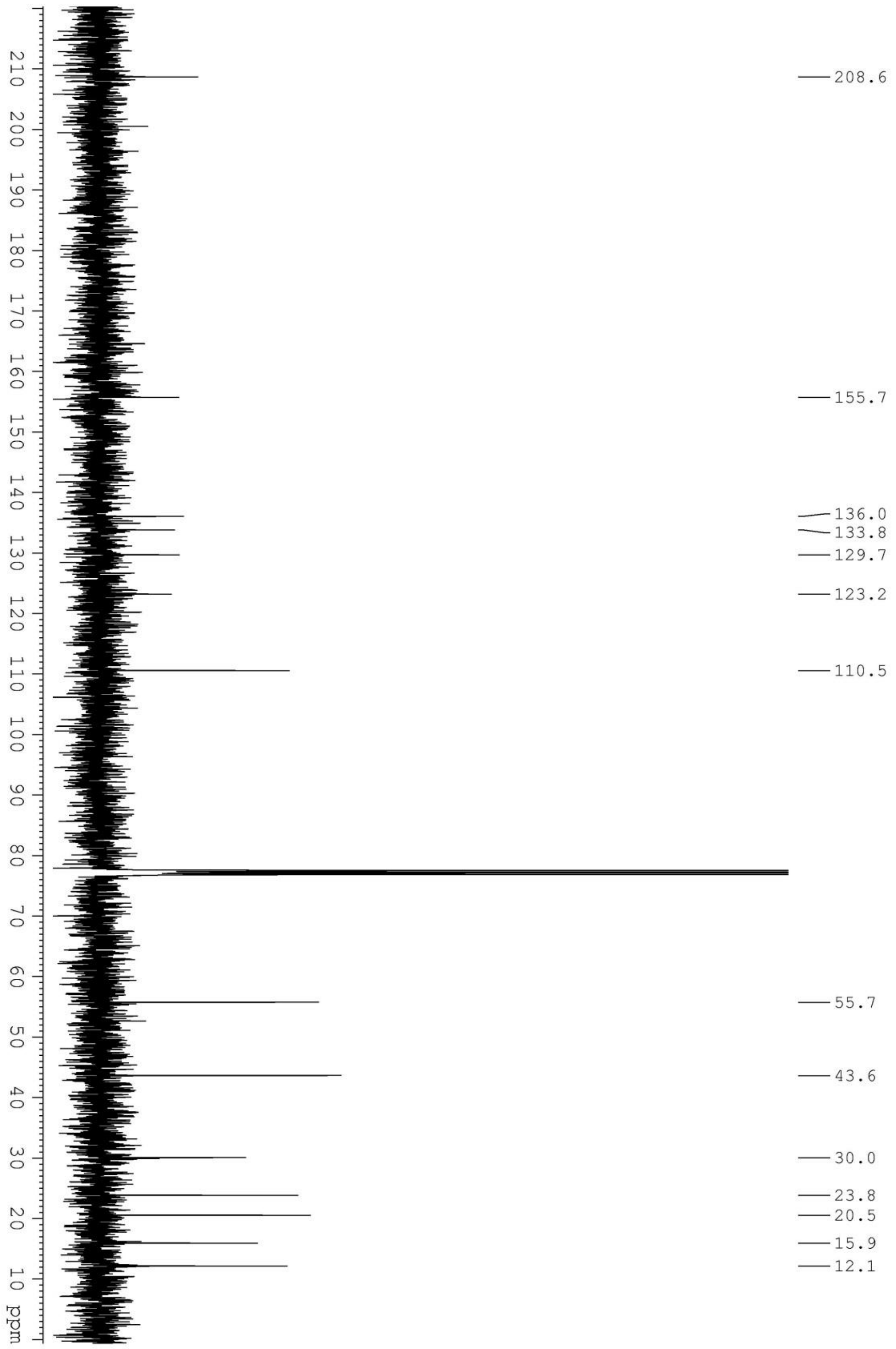

Figure 12: ^{13}C NMR of **13** ($CDCl_3$, 100 MHz).

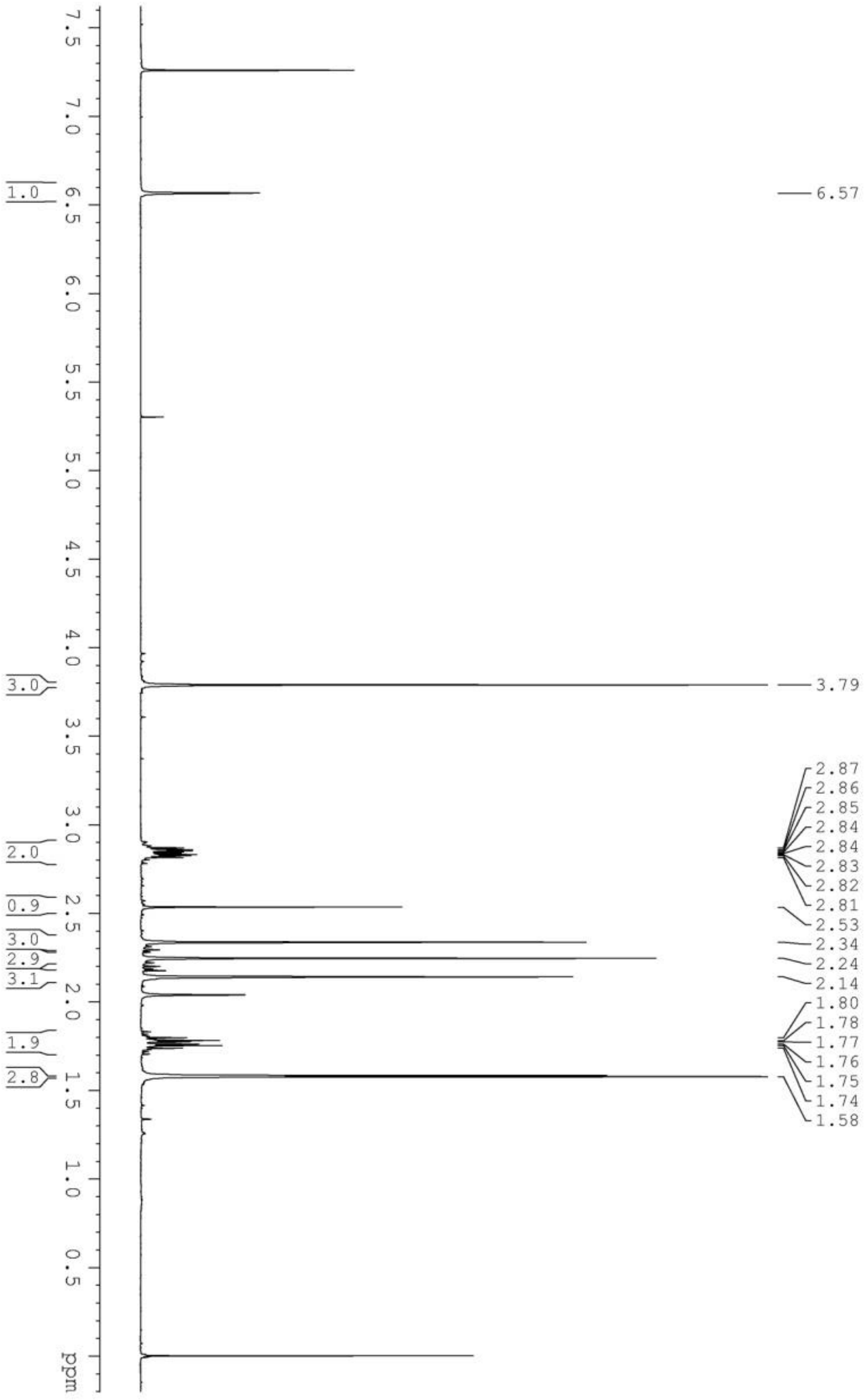

Figure 13: ^{1}H NMR of **9** ($CDCl_3$, 400 MHz).

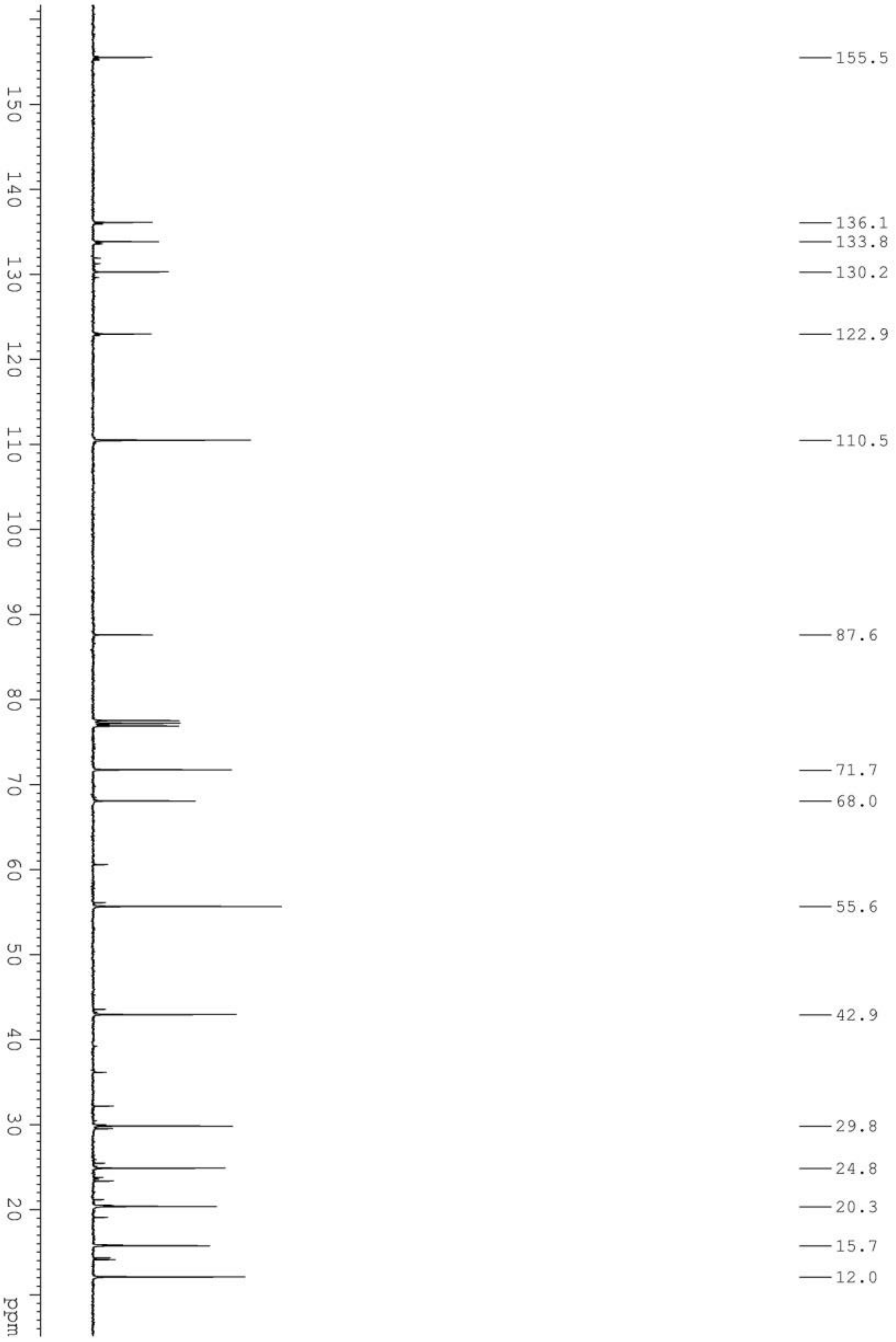

Figure 14: ^{13}C NMR of **9** ($CDCl_3$, 100 MHz).

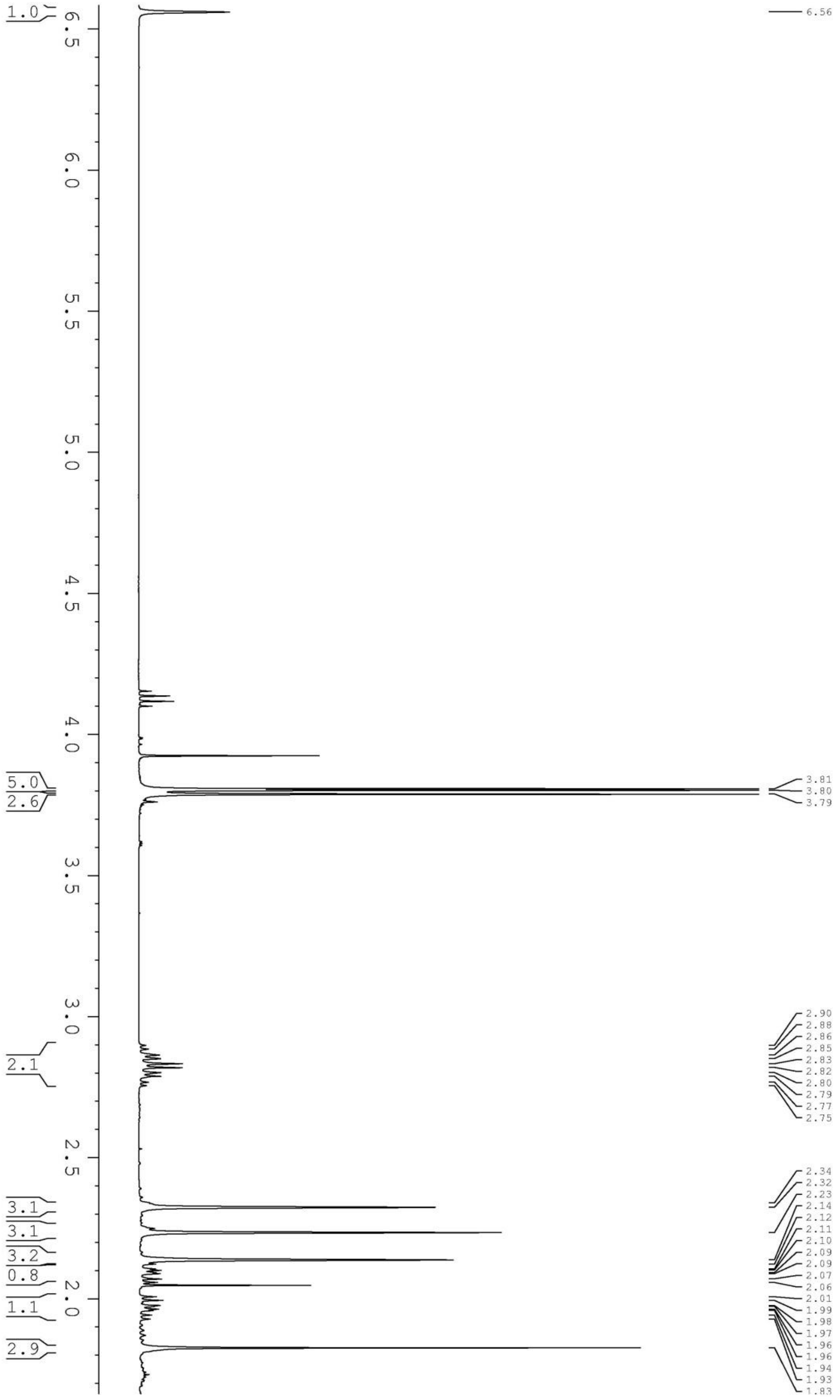

Figure 15: ^{1}H NMR of **19** ($CDCl_3$, 400 MHz).

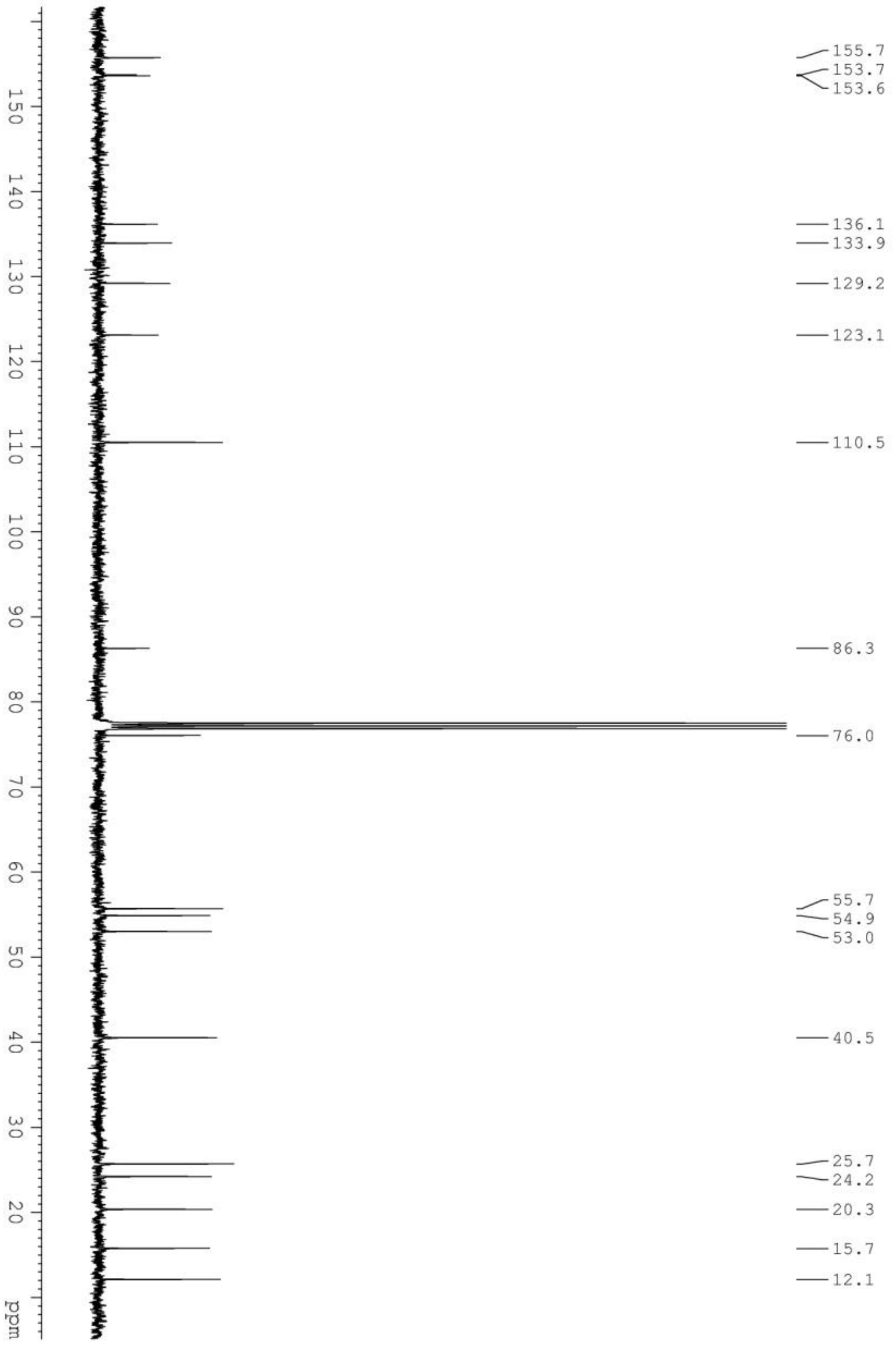

Figure 16: ^{13}C NMR of **19** ($CDCl_3$, 100 MHz).

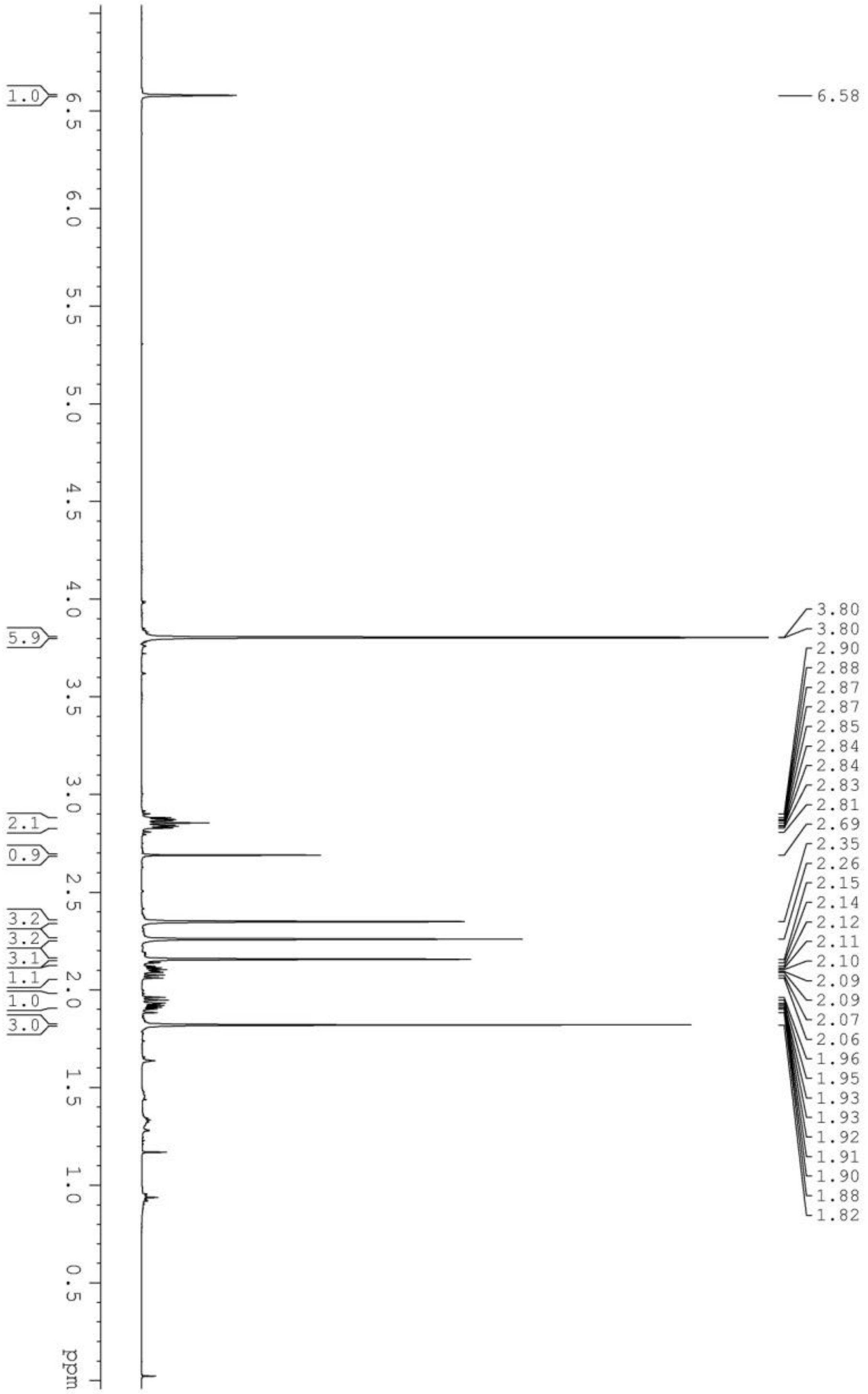

Figure 17: ^{1}H NMR of **18** ($CDCl_3$, 400 MHz).

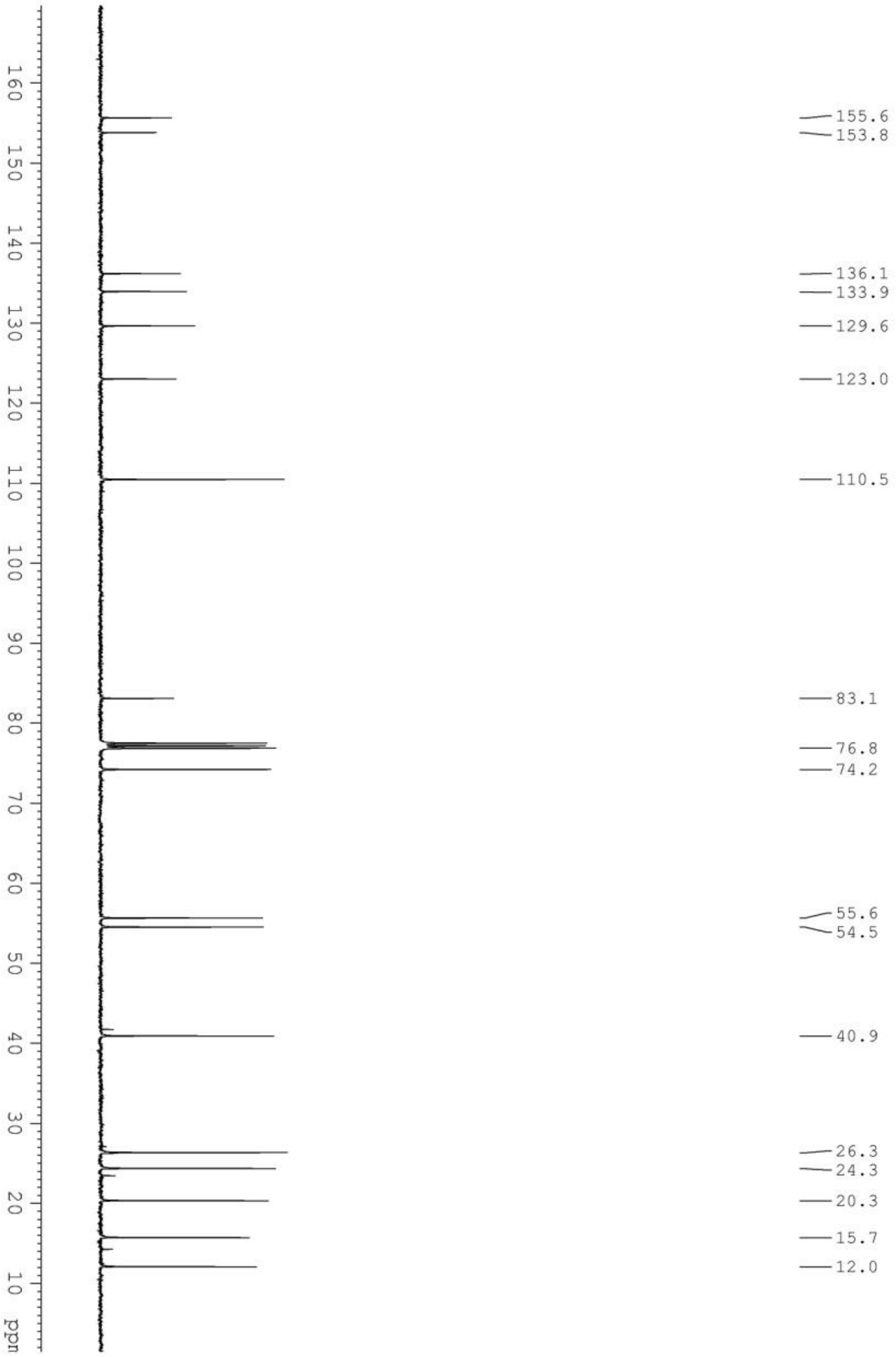

Figure 18: ^{13}C NMR of **18** ($CDCl_3$, 100 MHz).

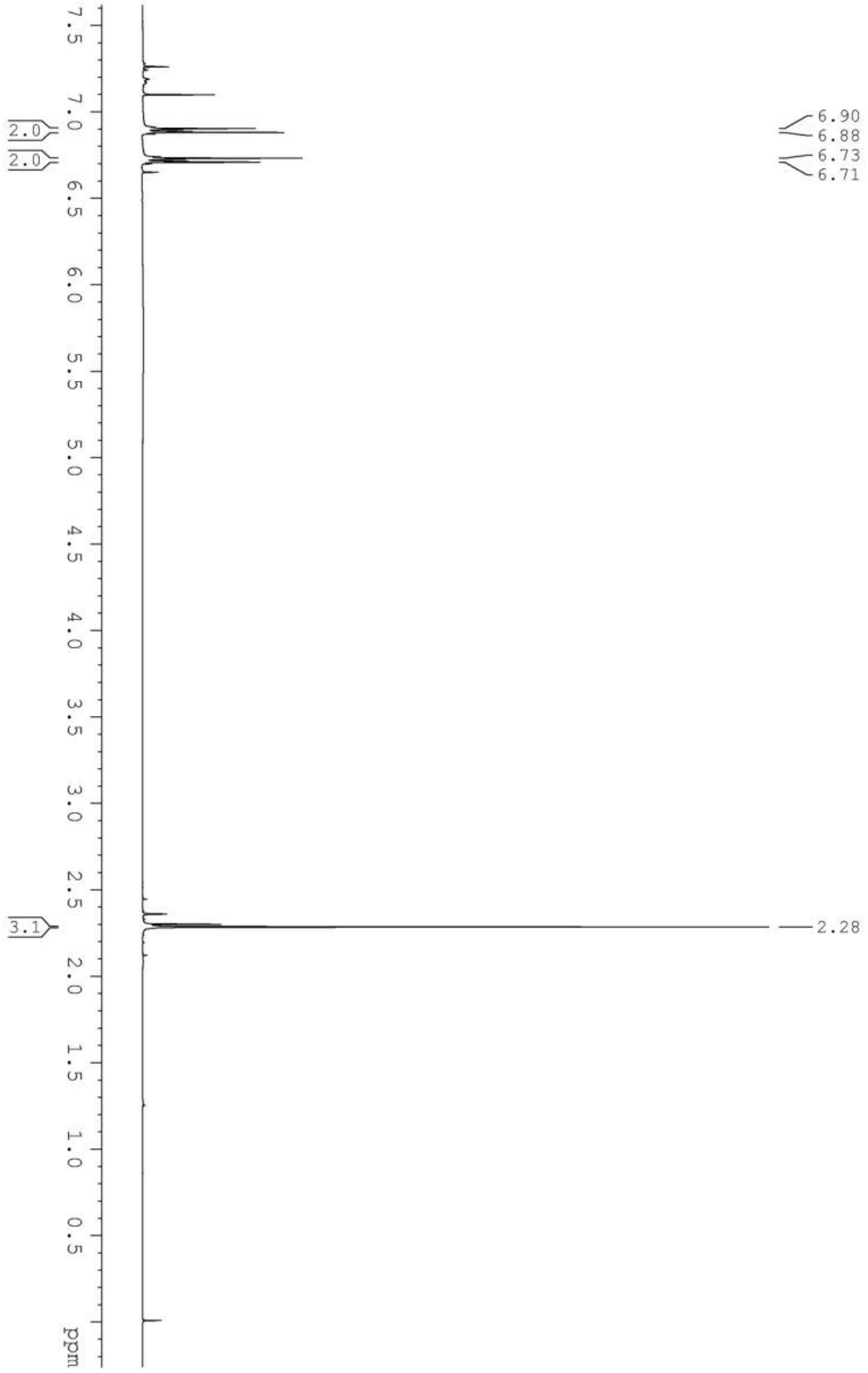

Figure 19: ^{1}H NMR of **16** ($CDCl_3$, 400 MHz).

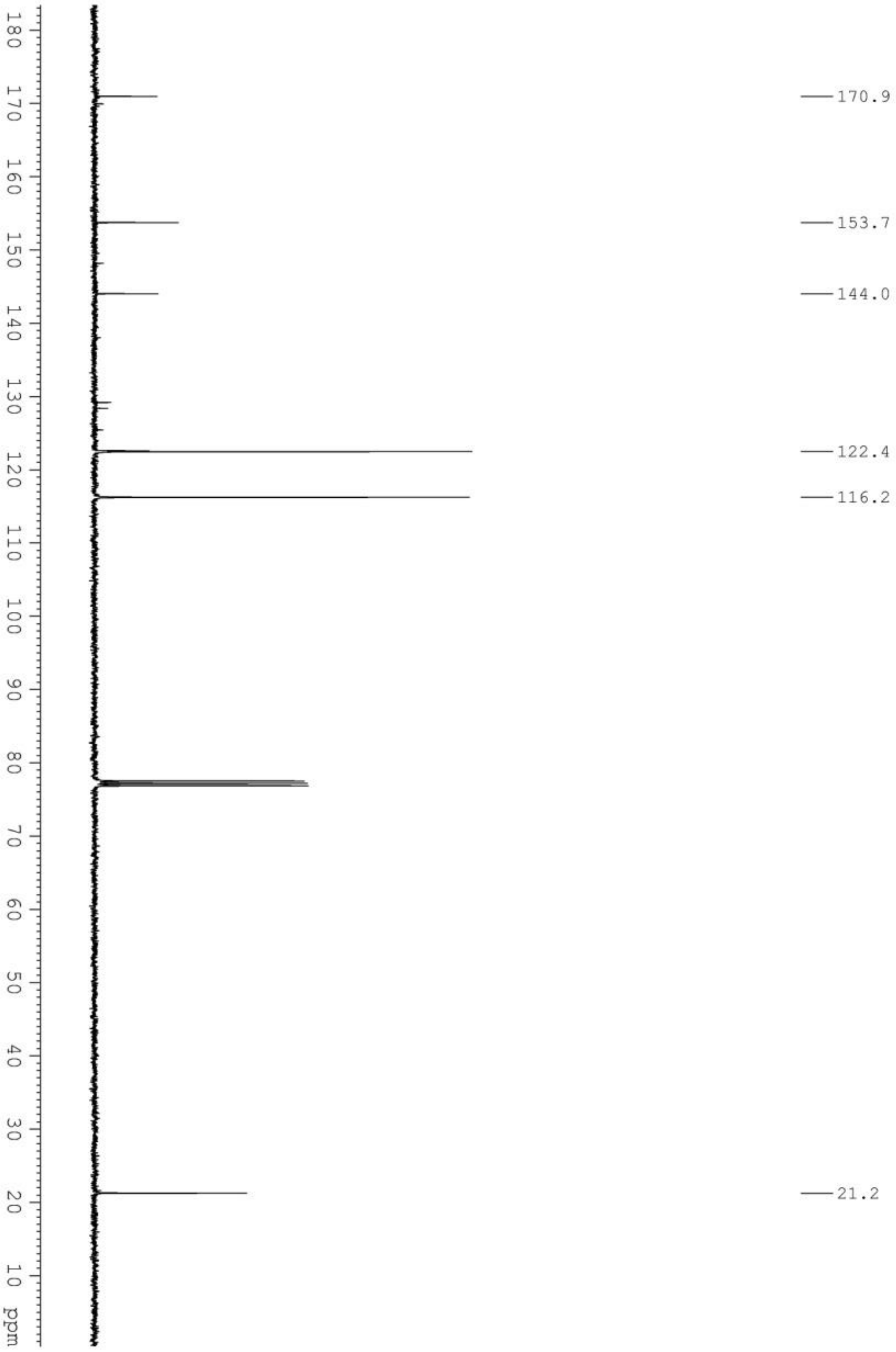

Figure 20: ^{13}C NMR of **16** ($CDCl_3$, 100 MHz).

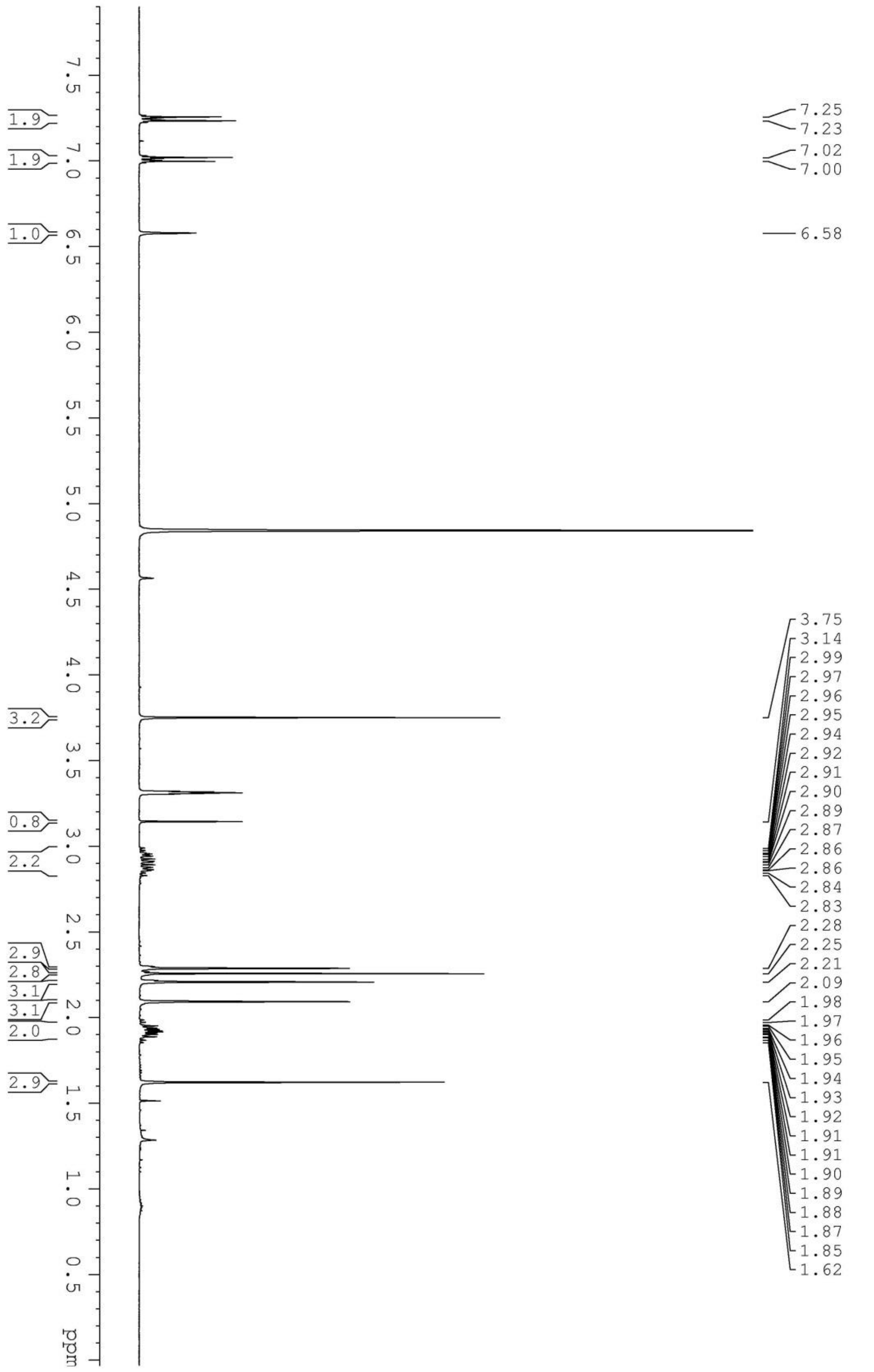

Figure 21: ^{1}H NMR of **8** (CD_3OD, 100 MHz).

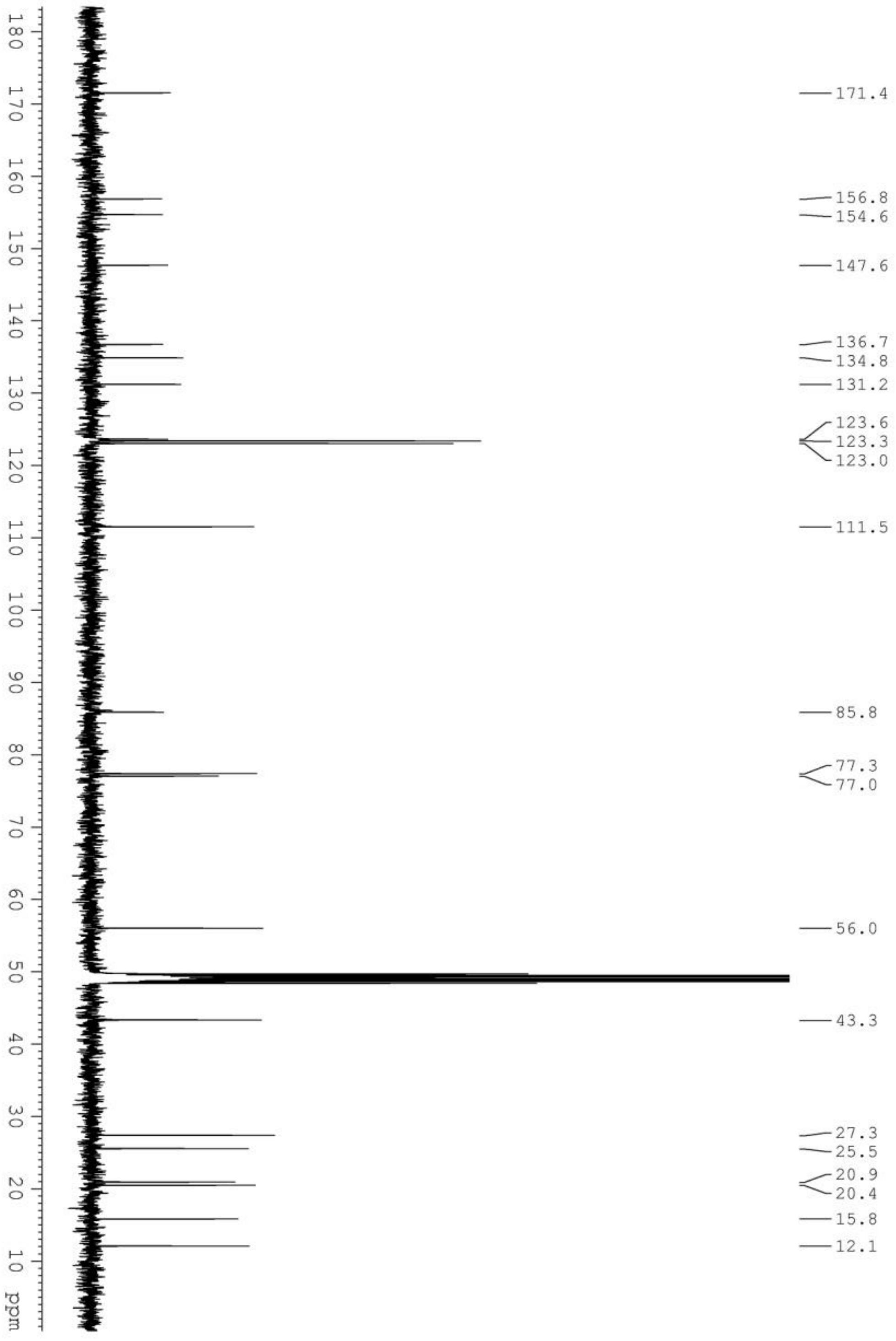

Figure 22: ^{13}C NMR of **8** (CD_3OD, 100 MHz).

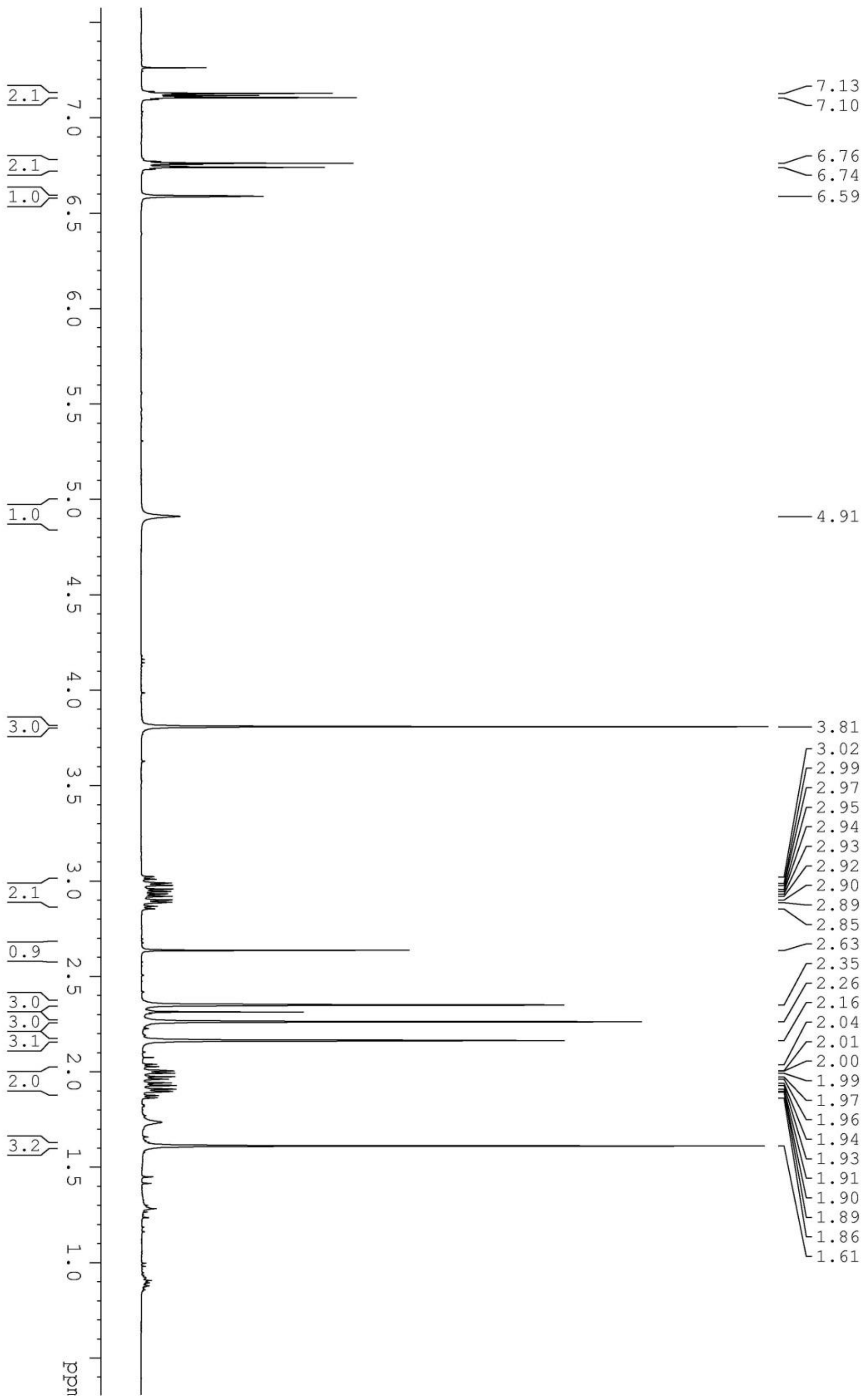

Figure 23: ^{1}H NMR of **20** ($CDCl_3$, 400 MHz).

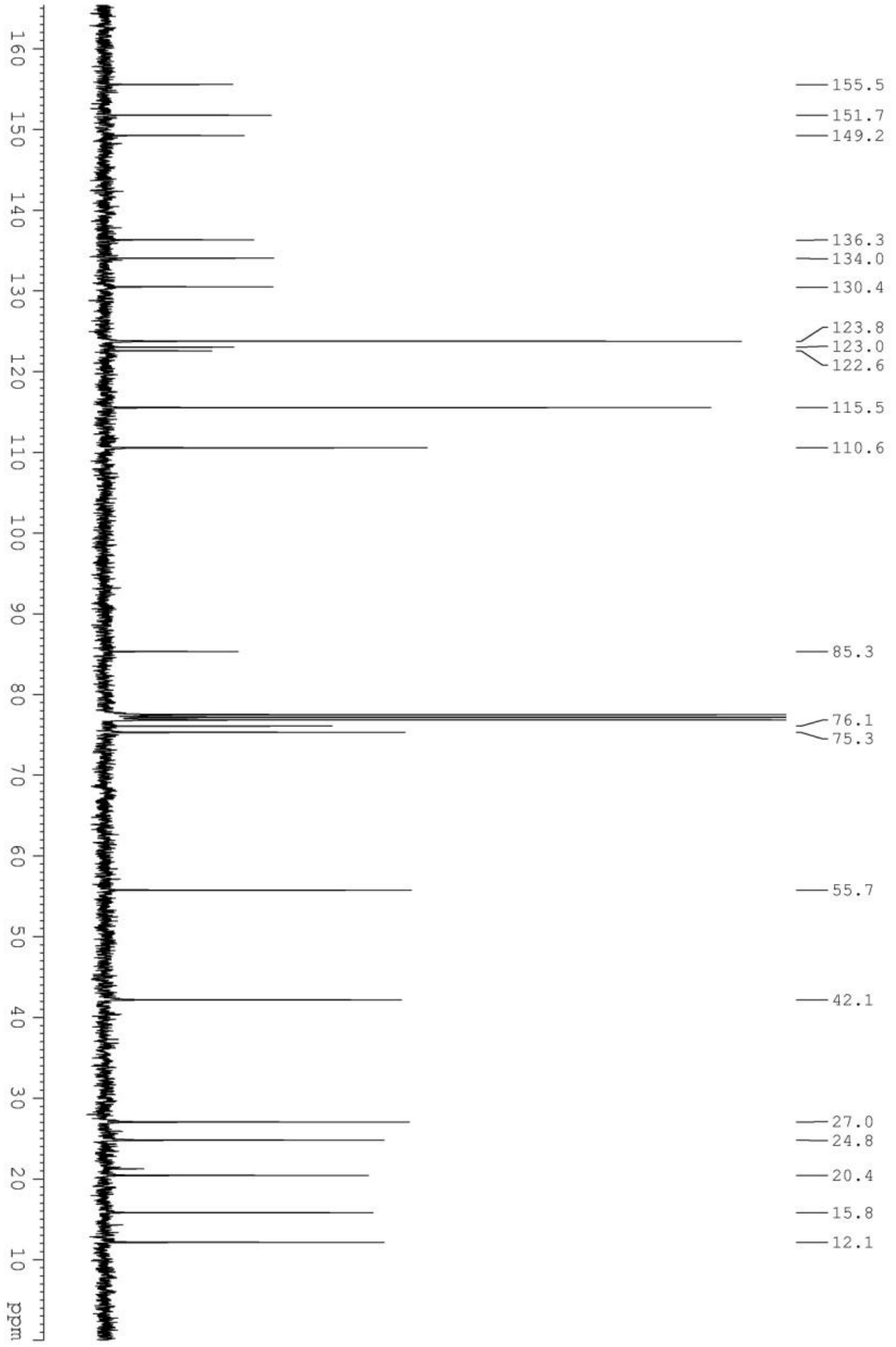

Figure 24: ^{13}C NMR of **20** ($CDCl_3$, 100 MHz).

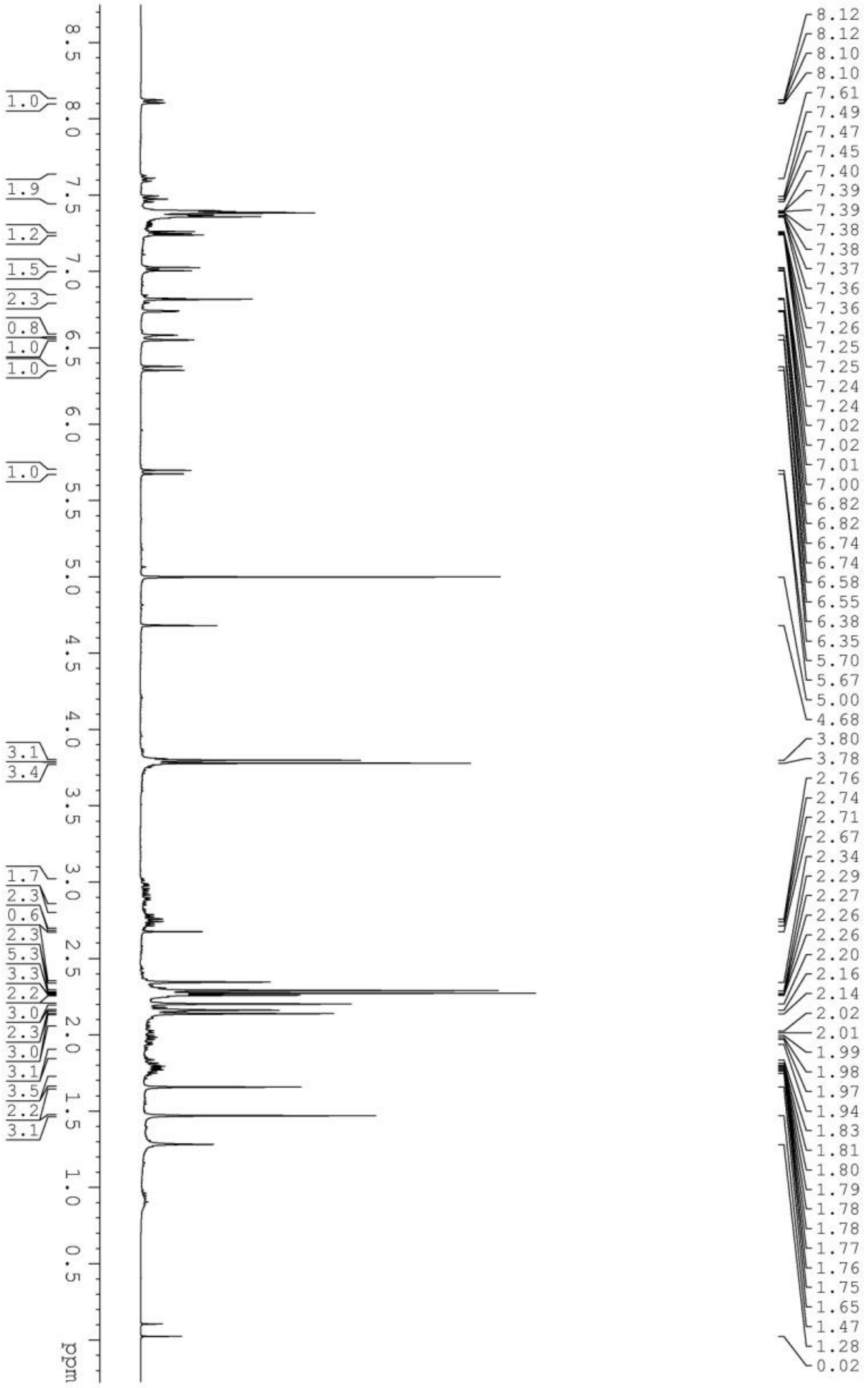

Figure 25: ¹H NMR of **20** and **21** ($CDCl_3$, 400 MHz).

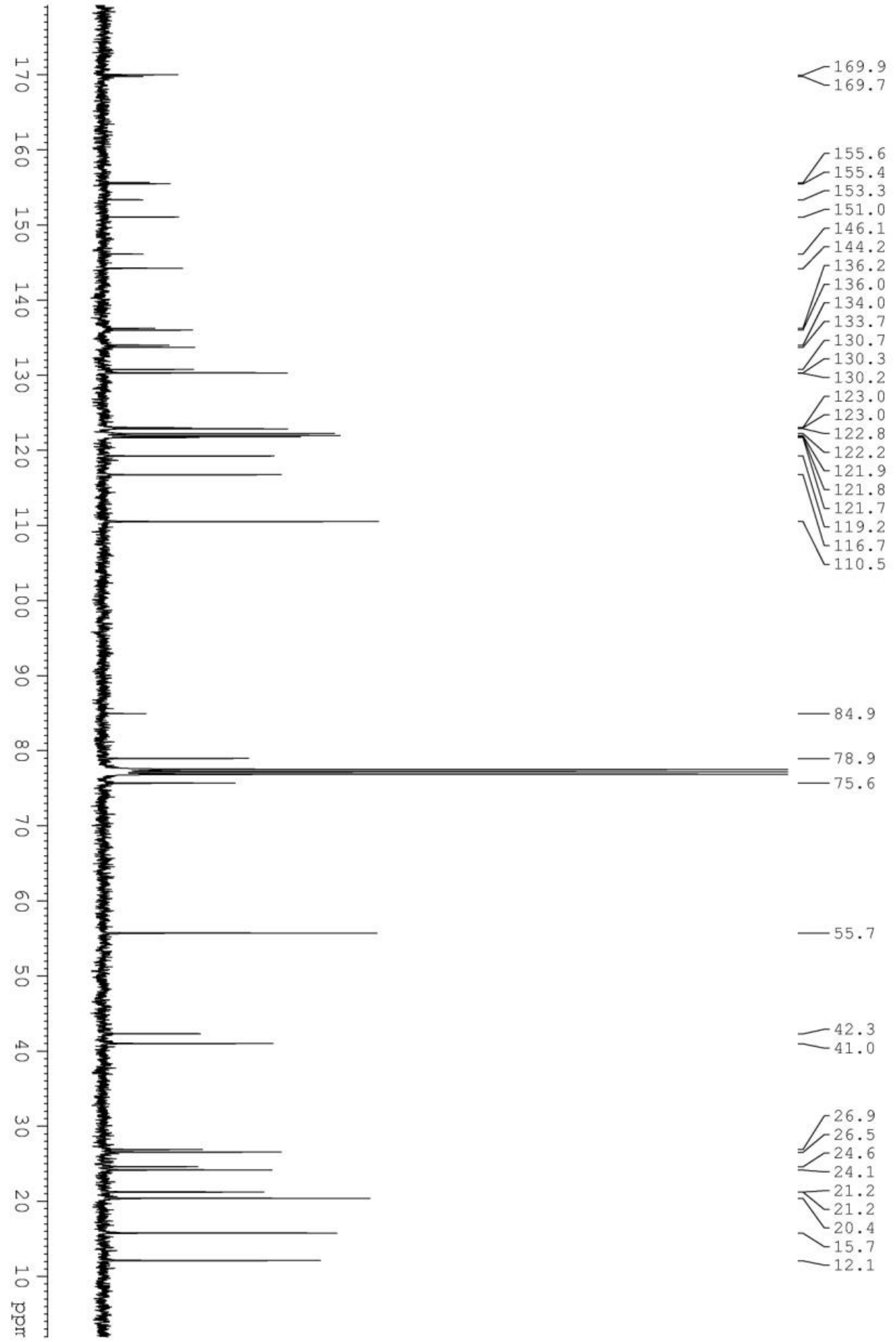

Figure 26: ^{13}C NMR of **20** and **21** ($CDCl_3$, 100 MHz).

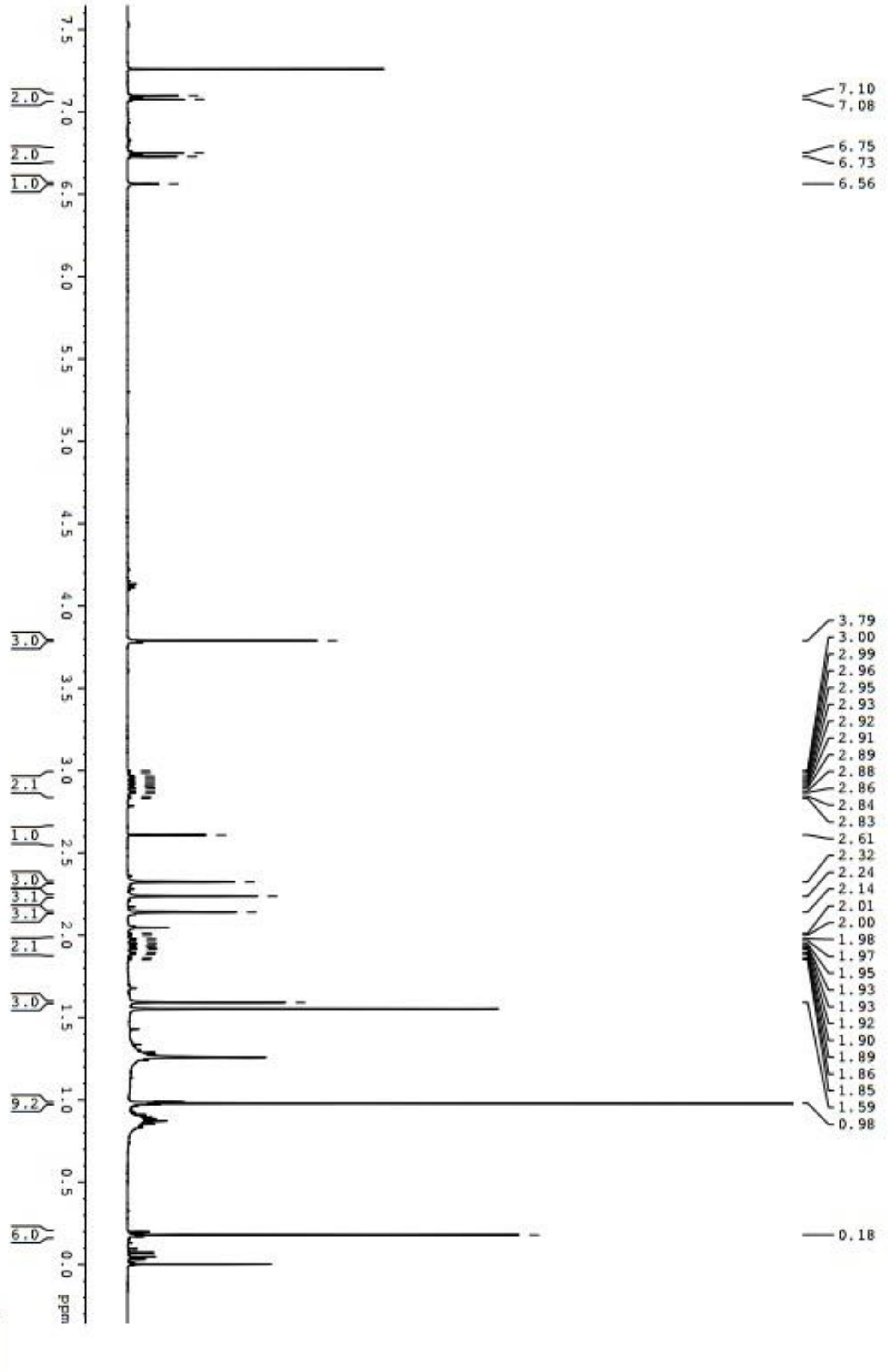

Figure 27: ^{1}H NMR of **22** ($CDCl_3$, 400 MHz).

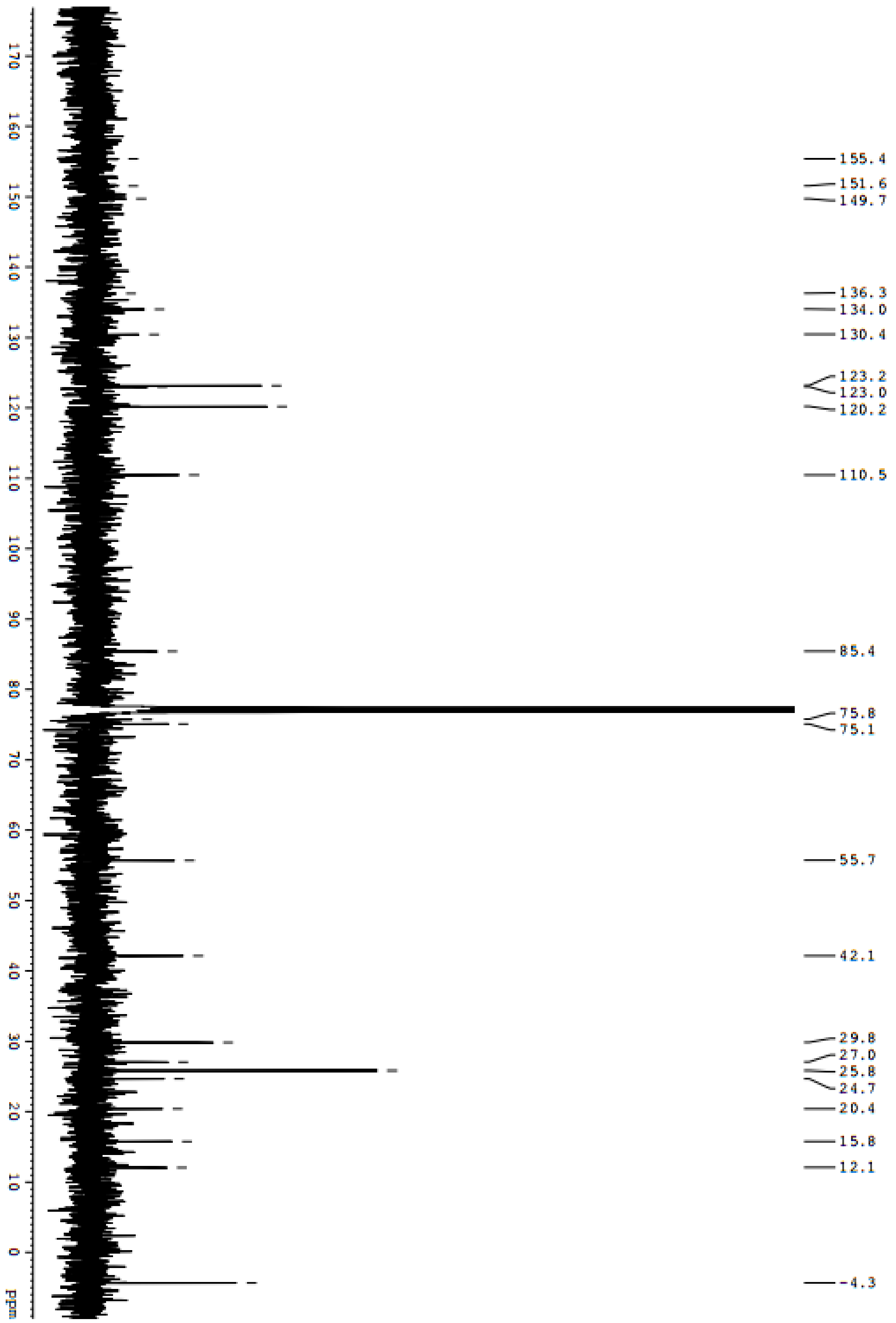

Figure 28: ^{13}C NMR of **22** ($CDCl_3$, 100 MHz).

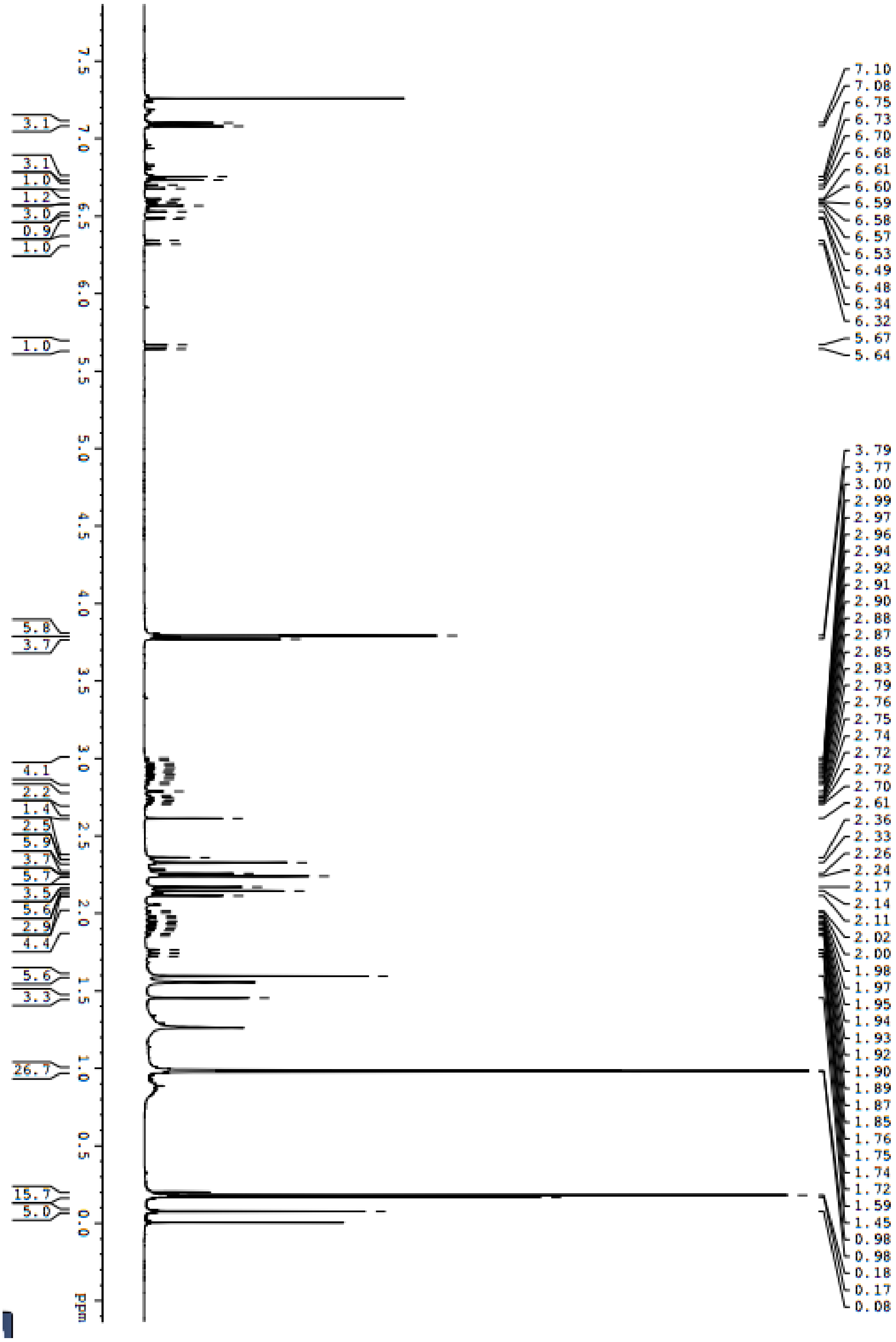

Figure 29: ^{1}H NMR of **22** and **23** ($CDCl_3$, 400 MHz).

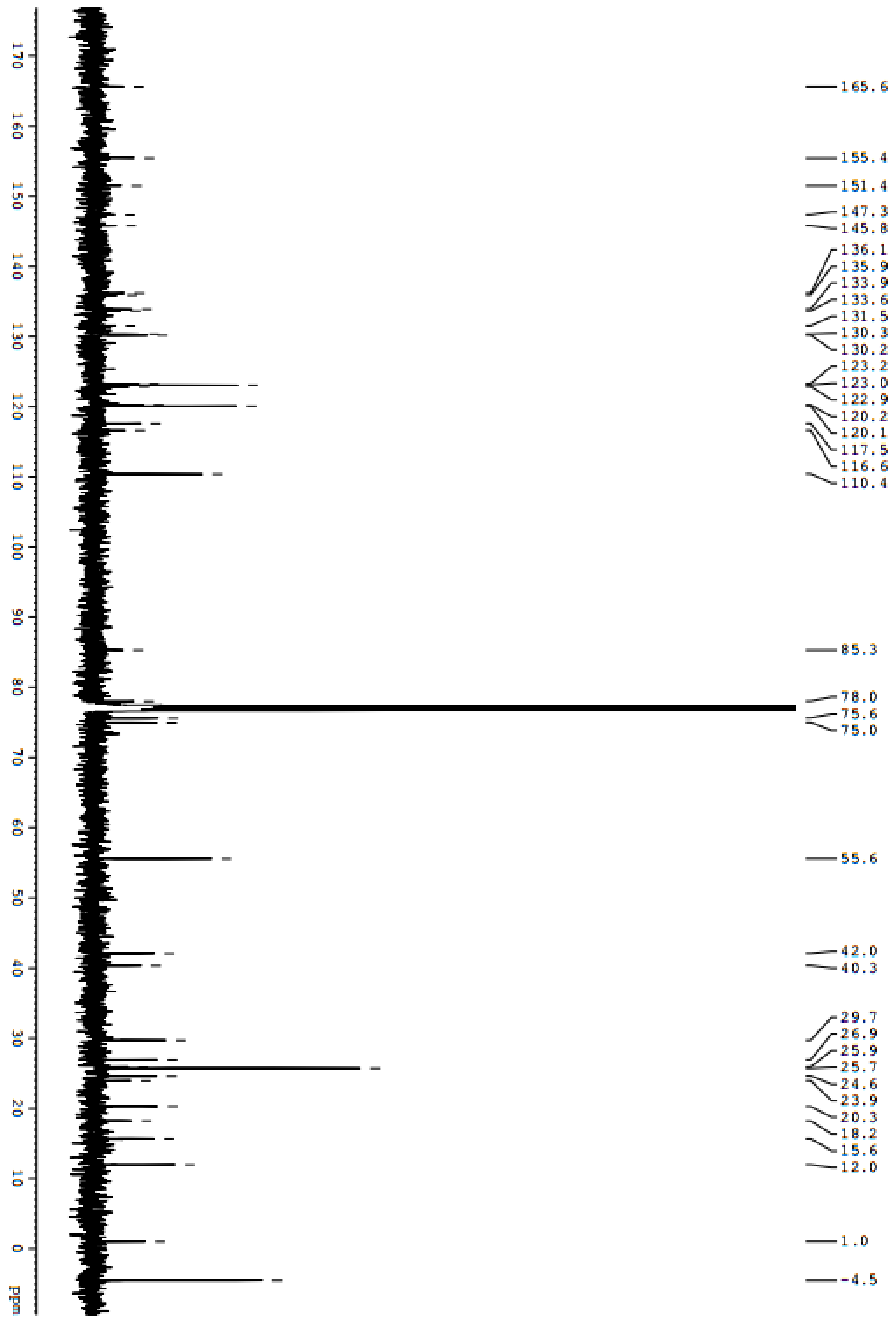

Figure 30: : ^{13}C NMR of **22** and **23** ($CDCl_3$, 100 MHz).

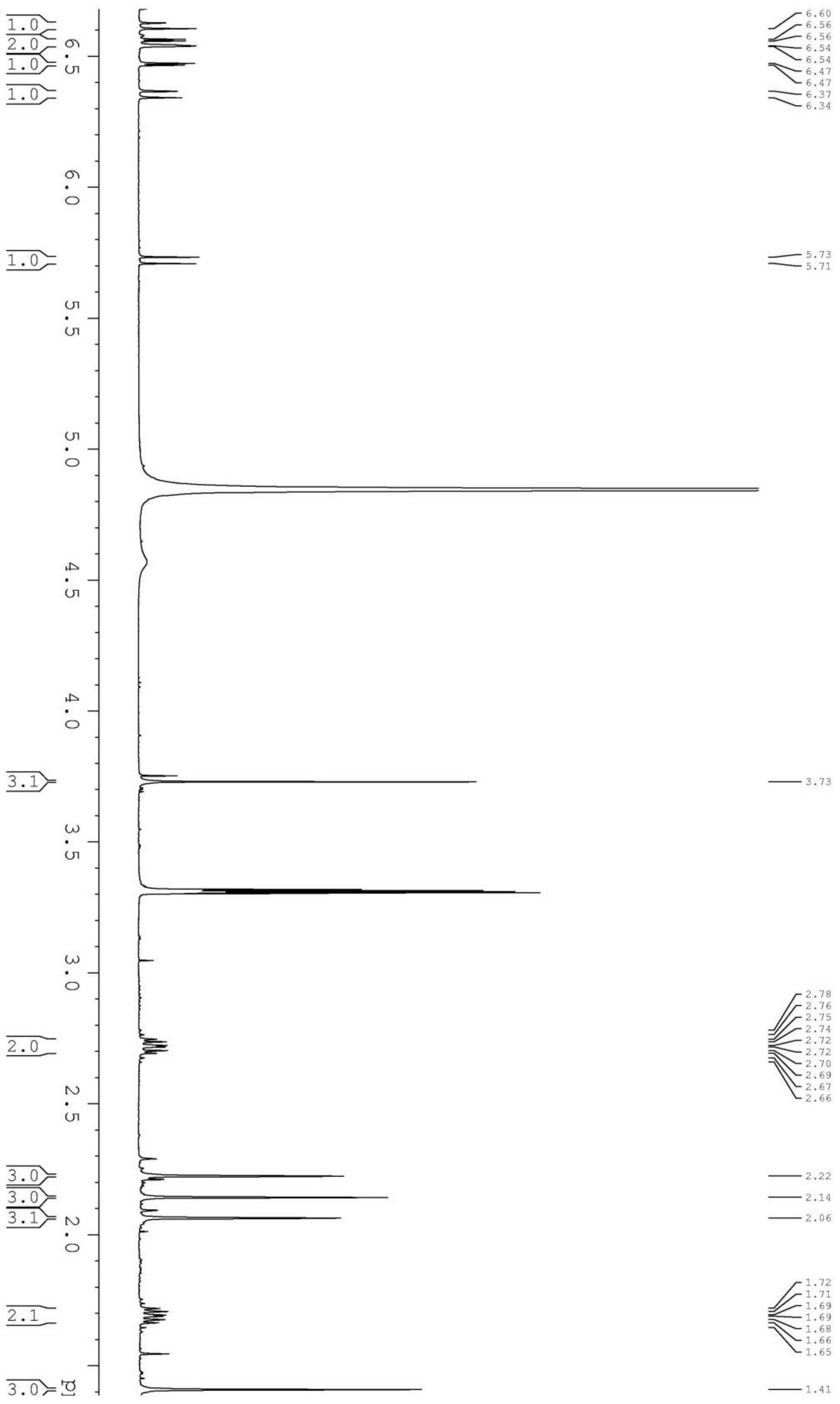

Figure 31: ^{1}H NMR of **5** (CD_3OD, 500 MHz).

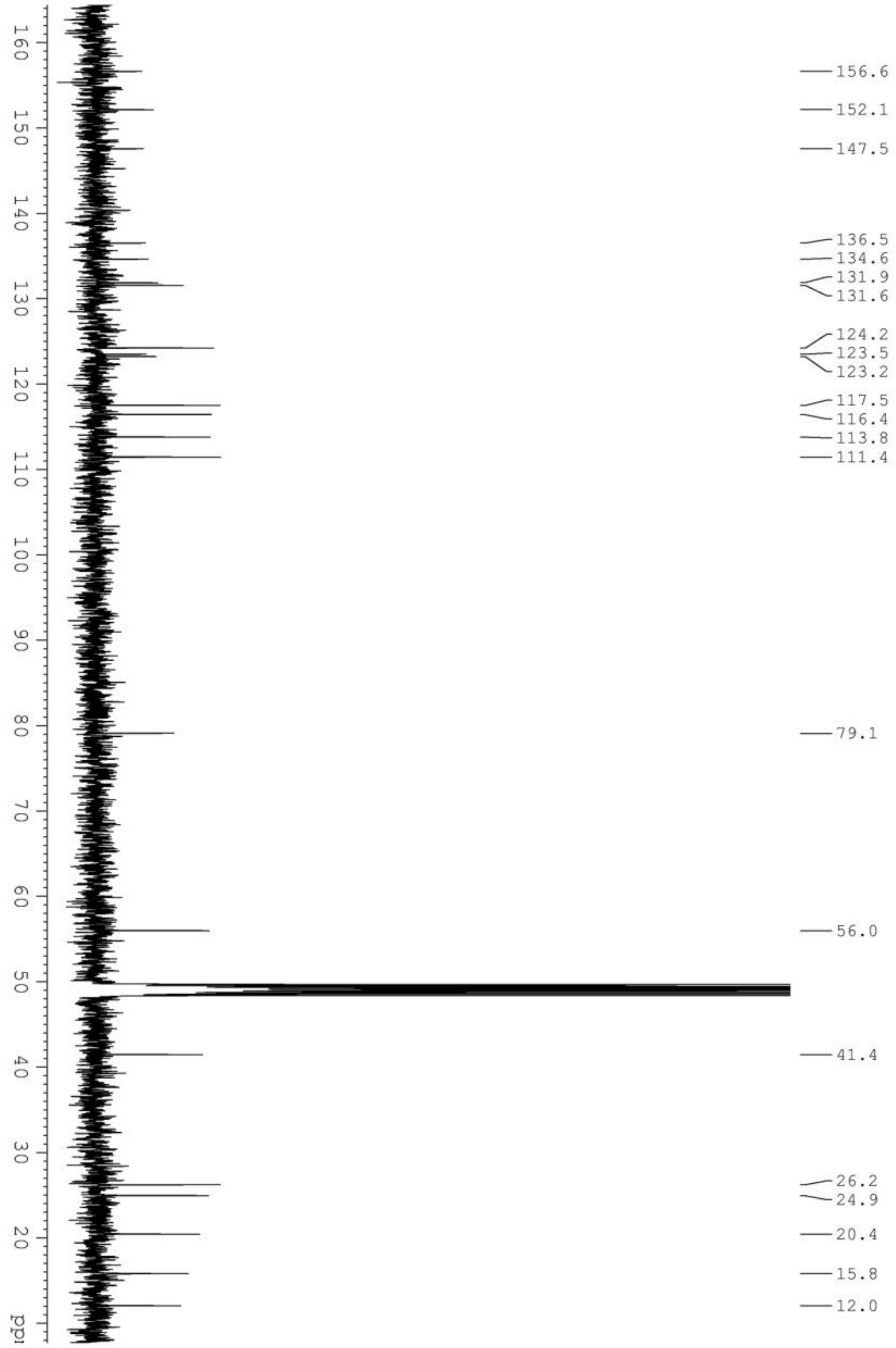

Figure 32: : ^{13}C NMR of **5** (CD_3OD, 125 MHz).
